# Regional variation in healthcare utilization among patients with depression in Germany: a multilevel analysis with PopGrouper-based multimorbidity adjustment

**DOI:** 10.1007/s43999-026-00092-6

**Published:** 2026-06-09

**Authors:** Anika Kreutzberg, Chrissa Tsatsaronis, Wilm Quentin, Reinhard Busse

**Affiliations:** 1 Department of Health Care Management, Technische Universität Berlin, Berlin, Germany; 2 Department for Planetary and Public Health, Universität Bayreuth, Bayreuth, Germany

**Keywords:** Depression, Socioeconomic inequalities, Regional analysis, Population classification, Claims data, Germany

## Abstract

**Background:**

Depression is associated with a substantial health and economic burden and shows pronounced regional variation in Germany. While socioeconomic and urban–rural differences in depression prevalence and service use are well documented, less is known about how regional socioeconomic deprivation and urbanization relate to healthcare utilization after accounting for multimorbidity.

**Methods:**

We analyzed nationwide claims data from a large German sickness fund including adults with diagnosed depression (*N* = 1,083,319). Healthcare utilization in 2023 was measured as healthcare costs, days of sickness absence, any outpatient mental health specialist contact, and outpatient psychotherapy use. Multilevel regression models with individuals nested within 96 spatial planning regions were estimated, adjusting for age, sex, and multimorbidity using the PopGrouper. Regional socioeconomic deprivation was measured using the German Index of Socioeconomic Deprivation, and urbanization was classified as urban, semi-urban, or rural. Proportional variance component analyses quantified explained regional heterogeneity.

**Results:**

Higher regional socioeconomic deprivation was significantly associated with more days of sickness absence (adjusted mean: 34.1 days in the least vs. 40.1 days in the most deprived regions) and a lower likelihood of receiving outpatient psychotherapy (17.9% vs. 16.3%). No significant associations were observed between deprivation and healthcare costs or mental health specialist contacts. Urban regions were associated with higher healthcare costs (2,284€ vs. 2,188€) and more frequent mental health specialist contacts (15.6% vs. 13.5%). Overall regional clustering was modest but most pronounced for mental health specialist contacts.

**Conclusion:**

Regional socioeconomic deprivation is linked to higher sickness absence due to depression. Regional inequalities in access to mental health services are small but largely unexplained by deprivation, urbanization, and multimorbidity.

**Supplementary Information:**

The online version contains supplementary material available at 10.1007/s43999-026-00092-6.

## Introduction

Depression is one of the leading contributors to disability worldwide and is associated with a substantial personal, social, and economic burden [1]. In Germany, depressive disorders account for considerable sickness absence and healthcare costs [2]. Previous research has demonstrated pronounced regional variation in depression prevalence, with higher rates in metropolitan areas and in southern and southwestern regions [3, 4]. Area-level socioeconomic deprivation and urbanization have been identified as important risk factors, with higher prevalence and symptom severity observed in socioeconomically disadvantaged and more semi-urban regions in Germany [3, 5, 6] and internationally [7, 8].

Despite the availability of effective treatments, depression care remains characterized by substantial treatment gaps. Population-based surveys and claims data analyses from Germany indicate that only a minority of individuals with depressive symptoms or diagnosed depression receive guideline-based psychotherapeutic or psychiatric care [9–12]. Utilization of mental health services varies markedly across regions and is associated with individual socioeconomic characteristics, age, and sex [9, 11, 13–15]. These inequalities persist despite the absence of direct financial barriers, suggesting that non-monetary factors such as provider availability, waiting times, and system navigation play an important role. In addition, lower socioeconomic status is associated with longer duration of sickness absence, while depression-related healthcare costs vary substantially by socioeconomic and demographic factors [16–18].

Overall, existing evidence suggests that regional socioeconomic and contextual characteristics shape both mental health burden and access to care. However, few studies have quantified the extent to which regional deprivation and urbanization explain variation in depression-related healthcare utilization, particularly after accounting for multimorbidity.

Against this background, the present study examines the association between regional socioeconomic deprivation, urbanization, and multiple indicators of depression-related healthcare utilization in Germany, including healthcare costs, sickness absence, any outpatient mental health specialist contact, and outpatient psychotherapy use. Accounting for multimorbidity is essential for fair and meaningful assessment of regional differences since individuals with depression have a high burden of both psychiatric and somatic comorbidities [19, 20]. While many studies adjust for age and sex, fewer account for comorbidities. Using nationwide claims data and multilevel regression models, we account for multimorbidity using the PopGrouper, a population-based classification system capturing comprehensive healthcare needs. Applying the PopGrouper for regional comparisons was introduced by Kreutzberg et al. [21] and tested in a cohort of diabetes patients by Tsatsaronis et al. [22]. This paper demonstrates another application of the PopGrouper for the assessment of regional health care variation. By combining multilevel modeling with variance decomposition, this study aims to provide a more nuanced understanding of regional inequalities in depression care and to inform region-sensitive health services planning.

## Methods

### Data and measures

The analysis is based on pseudonymized claims data from the BARMER sickness fund. BARMER covers approximately 10% of the German population and is represented in all federal states. The data include information on demographics and the full spectrum of inpatient and outpatient services, rehabilitation, prescription drugs, remedies, and medical aids. The study follows a longitudinal design using 2022 data to define the study population and baseline morbidity, and 2023 data to assess healthcare utilization indicators. This temporal separation was chosen to avoid simultaneity between morbidity and outcomes.

#### Study population

The study population comprised adults aged ≥ 18 years with diagnosed depression in 2022 (baseline year) who were continuously insured in 2022 and either continuously insured or deceased in 2023. Depression diagnoses were identified using the criteria of the German morbidity-based risk adjustment scheme (Morbi-RSA) [23], requiring confirmed outpatient diagnoses in at least two quarters of 2022. Additional medication and treatment-duration criteria applied to severe and recurrent depression. Diagnostic codes and selection criteria are provided in Supplement A.

#### Healthcare utilization

Four indicators related to healthcare utilization were analyzed for 2023 (follow-up year): (1) total healthcare costs, defined as the sum of all expenditure related to inpatient, outpatient, rehabilitative, pharmaceutical, remedies, medical aids, and midwifery services; (2) days of certified sickness absence, measured for individuals aged ≤ 67 and restricted to those with at least one day of absence; (3) any outpatient mental health specialist contact, defined as at least one outpatient depression-related contact with a psychotherapist (including both physician psychotherapists and psychological psychotherapists) or psychiatrist (according to physician specialty classifications assigned by the Association of Statutory Health Insurance Physicians [24]); and (4) use of outpatient psychotherapy services, defined as use of at least one outpatient psychotherapy service reimbursed under the German Psychotherapy Guideline [25] (Chap. 35 of the Uniform Value Scale (EBM)). A small number of negative cost values were set to zero, and healthcare costs and sickness absence days were log-transformed due to right-skewed distributions.

#### Patient demographics and PopGroup-morbidity

Age was grouped into five categories (18–39, 40–59, 60–69, 70–79, ≥ 80), and sex was classified as female or male/other. Multimorbidity was accounted for using the PopGrouper version 1.0 based on diagnoses and healthcare utilization in 2022 (baseline year). The grouping algorithm is published by Braun et al. [26]. PopGrouper 1.0 comprises 776 PopGroups which can be aggregated to 10 Macro PopGroups which reflect broad population segments defined by medical characteristics and resource use (e.g., newborns, pregnancy, or severe high-cost cases). To ensure robust estimation, PopGroups representing at least 1% of the study population were included directly; smaller groups were aggregated to their respective Macro PopGroups, resulting in 19 (Macro)PopGroups. Individuals assigned to the pregnancy-related Macro PopGroup (*n* = 8,520; <0.8%) were excluded.

#### Regions and regional characteristics

Individuals were assigned to one of 96 German spatial planning regions based on residence in 2022 (baseline year) [27]. Aggregation from district to spatial planning regions was necessary to ensure sufficient case numbers by age, sex, and morbidity group in each region. Regional socioeconomic deprivation was measured using quintiles of the German Index of Socioeconomic Deprivation (GISD from 2019) developed by the Robert Koch Institute [28]. The GISD serves as a measure of relative regional socioeconomic deprivation including the dimensions occupation, education, and income, and ranges between 0 (lowest deprivation) and 1 (highest deprivation). Level of urbanization was classified using the regional typology based on settlement structure in 2022 [29]. The typology distinguishes urban regions (≥ 50% of the population living in major or medium-sized cities and high population density), semi-urban regions (33–49% urban population and intermediate density), and rural regions (< 33% urban population and low population density).

### Analyses

#### Descriptive analyses

We describe regional variation using maps and PopGroup standardized outcome ratios (PGSOR) as proposed and detailed by Kreutzberg et al. [21]. The PGSOR represents the ratio of the observed number of events for a given outcome variable in a region and the expected number of events according to the region’s PopGroup composition. It serves as a measure of relative regional performance compared to the national average after standardizing for the PopGroup distribution in each region.

#### Multilevel regression models

Associations between regional deprivation, urbanization, and healthcare utilization were analyzed using multilevel regression models with individuals nested within regions as introduced by Kreutzberg et al. [21]. The continuous and log-transformed outcomes were modelled using linear mixed-effects models. The binary outcomes were analyzed using mixed-effects logistic regression. All models included random intercepts at the regional level and were estimated sequentially: an empty (null) model (M0), models adjusted for deprivation (M1DEP) or urbanization (M1URB) only, fully adjusted models additionally including age, sex, and (Macro)PopGroups (M2DEP, M2URB). A combined model including both deprivation and urbanization next to age, sex, and (Macro)PopGroups with and without interacting deprivation and urbanization was tested as well. Model fit comparisons indicated that the additive and interaction models did not consistently improve model fit, suggesting limited incremental value of combining both contextual indicators. Supplement B provides comparative model fit statistics.

#### Proportional variance component analysis

We applied a proportional variance component analysis to assess how much of the between-region variance was explained by regional and individual-level covariates. Between-region variance was quantified using intra-class correlation coefficients (ICC) and proportional change in variance (PCV) relative to the null model, following Kreutzberg et al. [21]. Positive PCV values indicate a reduction in between-region variance compared with the null model, whereas negative values indicate an increase in regional variance after adjustment.

All analyses were performed using SAS Enterprise Guide 8.4.

## Results

### Descriptive results

#### Study population and PopGroup morbidity

The study population included 1,083,319 individuals. Table 1 presents the composition of the study population in terms of demographic and regional characteristics. The majority (71.6%) were female, the largest age group (30.0%) consists of individuals aged 40–59 years. More than 47.7% of insured individuals in the study population live in urban areas, and the largest group (26.7%) comes from regions with medium socioeconomic deprivation.

**Table 1:** Demographic and regional characteristics of study population

Demographics	*n* (%)		Regional characteristics	*n* (%)	
**Total**	1,083,319	(100.0)	Quintiles of socioeconomic deprivation*		
**Sex**			Lowest deprivation	224,588	(20.7)
Male	308,160	(28.5)	Second lowest	221,655	(20.5)
Female	775,159	(71.6)	Medium deprivation	289,087	(26.7)
**Age group**			Second highest	180,959	(16.7)
Age 18–39	131,323	(12.1)	Highest deprivation	167,030	(15.4)
Age 40–59	324,940	(30.0)	Level of urbanization**		
Age 60–69	261,826	(24.2)	Urban region	516,550	(47.7)
Age 70–79	183,549	(16.9)	Intermediate urbanization	315,889	(29.2)
Age > = 80	181,681	(16.8)	Rural region	250,880	(23.2)

Note: * based on German Index of Socioeconomic Deprivation in 2019, ** 2022

The study population was divided into 672 PopGroups which were used to calculate the PGSOR. For the regression analyses, PopGroups with a prevalence of less than 1% were aggregated into Macro PopGroups, resulting in 19 (Macro)PopGroups that each accounted for at least 1% of the study population. Overall, 37% of patients were classified into 14 non-aggregated PopGroups, whereas the remaining 63% were represented by five higher-level aggregated Macro PopGroups. Tables 2 and 3 lists the prevalences of these 19 (Macro)PopGroups. The most prevalent PopGroup (9.7%) was P07060BB “Mild or moderate depression + at most 6 MDGs and age ≥ 25 years”. The remaining PopGroups demonstrate the high prevalence of other comorbidities in this population.

**Table 2:** PopGroups used for regression analyses with prevalence in 2022 (baseline year)

PopGroup	PopGroup name	*N*	%
P07060BB	P07: Mild or moderate depression + at most 6 MDGs and age ≥ 25 years	105,461	9.7
P06023BB	P06: Severe depression + at most 14 MDGs and age ≥ 18 years	77,066	7.1
P05106BB	P05: Other (very) severe condition(s) + at most 13 MDGs without long-term insulin treatment	29,969	2.8
P06034BB	P06: Neurological diseases (MDG) & Chronic pain + at most 15 MDGs without long-term insulin treatment	26,156	2.4
P07025BZ	P07: Mild or moderate depression & Osteoarthritis or osteochondrosis + at most 9 MDGs	22,901	2.1
P07052ZB	P07: Phobic, panic or anxiety disorder and age ≥ 25 years	22,820	2.1
P06045BB	P06: Diabetes mellitus with disease symptoms affecting at least one organ system + at most 14 MDGs and level of care dependency ≤ 2	20,484	1.9
P06063BB	P06: Diseases of the lungs (MDG) & Diseases of the heart (MDG) + at most 12 MDGs and level of care dependency ≤ 2	18,192	1.7
P07042BB	P07: Chronic pain + at most 9 MDGs and level of care dependency ≤ 1	16,954	1.6
P07010BB	P07: Arthritis + at most 11 MDGs and level of care dependency ≤ 1	14,650	1.4
P07050BB	P07: Short-duration mental disorder + at most 5 MDGs and age ≥ 25 years	12,013	1.1
P06087BB	P06: Other moderate condition(s) + at most 9 MDGs and age < 80 years	11,864	1.1
P06067BB	P06: Personality disorder + at most 7 MDGs and age ≥ 25 years	11,560	1.1
P06028BB	P06: Parkinson’s disease or other movement disorder + at most 13 MDGs and level of care dependency ≤ 3	11,212	1.0
**Total**		**401,302**	**37.0**

Note: MDG = Major Disease Group. The grouping algorithm is published by Braun et al. [26]

**Table 3:** Macro PopGroups used for regression analyses with prevalence in 2022 (baseline year)

Macro PopGroup	Macro PopGroup name	*N*	%
P03	Severe high-cost cases	51,396	4.7
P04	Actively treated malignant neoplasms	22,344	2.1
P05	At least one severe condition^1^	175,158	16.2
P06	At least one moderate condition^2^	260,524	24.0
P07	At least one mild condition^3^	172,595	15.9
**Total**		**682,017**	**63.0**

Note: ^1^ other than P05106BB, ^2^ other than P06023BB, P06028BB, P06034BB, P06045BB, P06063BB, P06067BB, P06087BB, ^3^other than P07010BB, P07025BZ, P07042BB, P07050BB, P07052ZB, P07060BB. The grouping algorithm is published by Braun et al. [26]

Mean healthcare costs per person in 2023 were 5,714€ (SD = 11,328). Days of sickness absence were measured for 66,259 individuals (8.4%) of the study population (aged 18–67, at least one day of absence). Mean number of days with sickness absence in 2023 was 85.5 (SD = 103.6). On average, 19.2% of individuals were treated at least once in 2023 by a psychotherapist and 17.7% received at least one outpatient psychotherapy service in 2023.

#### Descriptive regional variation

**Fig. 1:**
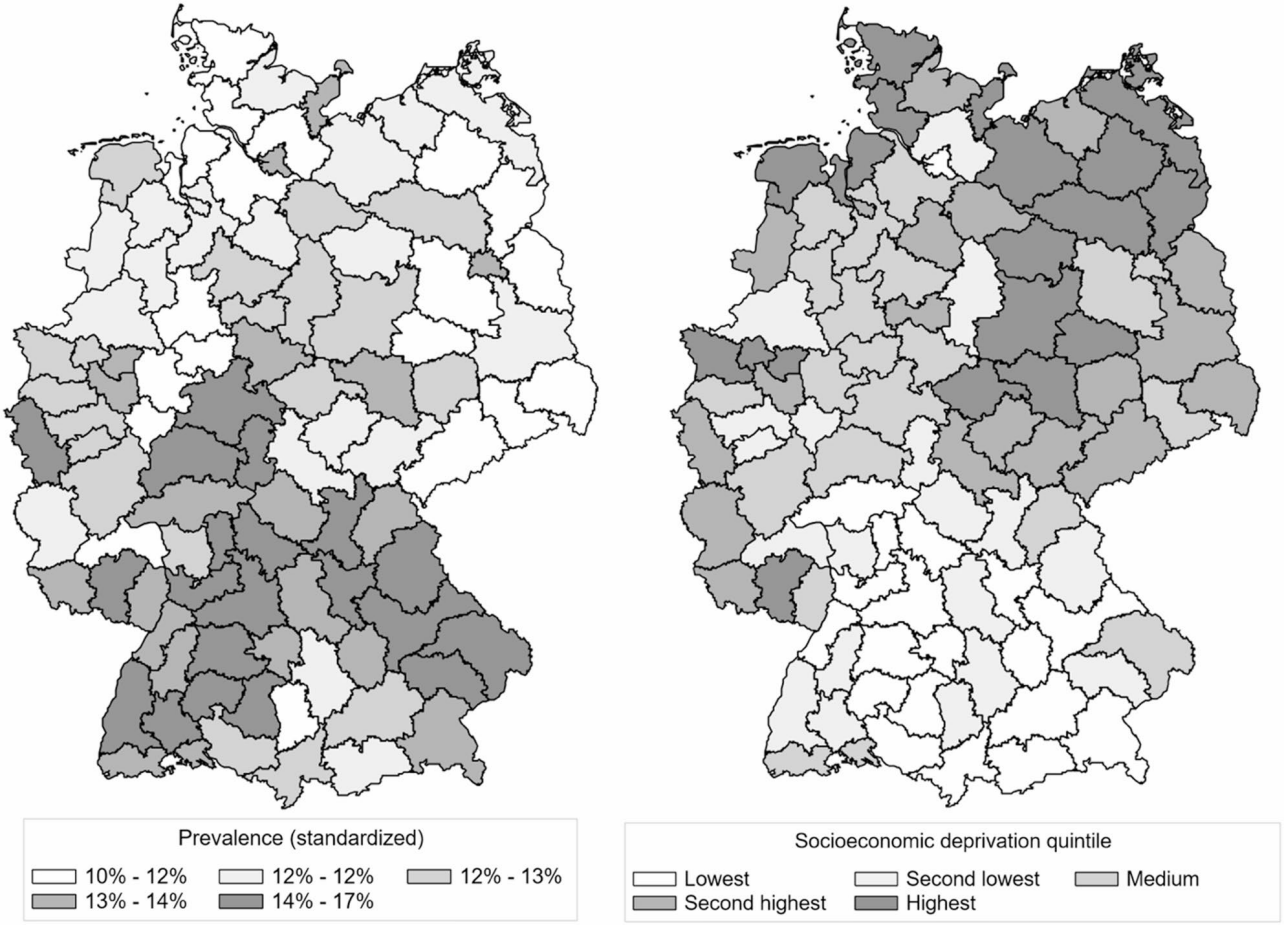
Regional distribution of claims-based prevalence of diagnosed depression in 2022 (age-sex-standardized) and socioeconomic deprivation based on GISD 2019

Figure 1 visualizes the regional distribution of age-sex-standardized claims-based prevalence of diagnosed depression and socioeconomic deprivation, with darker regions indicating higher prevalences and higher deprivation, respectively. Prevalence estimates refer to 2022, the baseline year used to define the study population. Regional prevalences range between 10.3% and 16.6%, with higher prevalences observed particularly in southern and southwestern Germany. The spatial distribution does not fully overlap with the regional pattern of socioeconomic deprivation. Several high-prevalence regions coincide with lower deprivation, and some more deprived regions also show elevated prevalence levels.

**Fig. 2:**
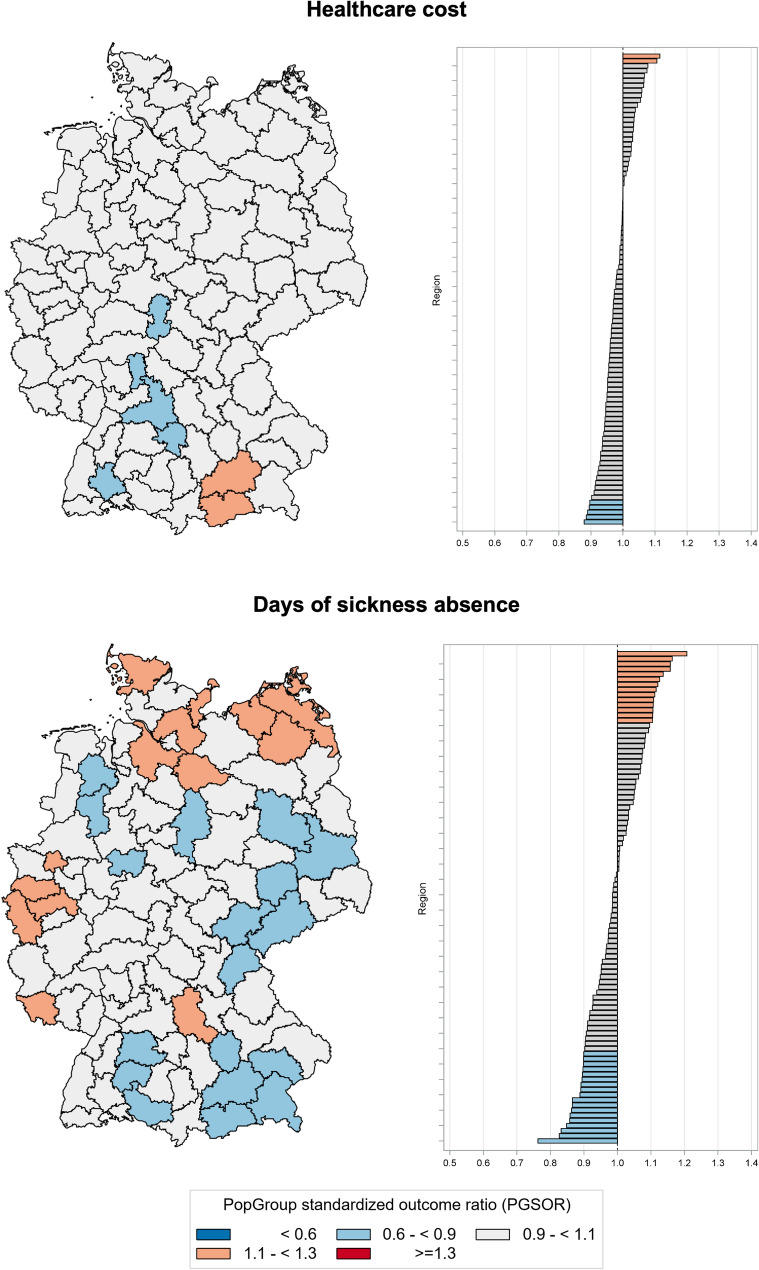
PopGroup standardized outcome ratios (PGSOR) in healthcare cost and days of sickness absence. Note: Red shades represent regions with larger than average, grey shades regions with approximately average, and blue shades regions with below average costs and days of sickness absence

**Fig. 3:**
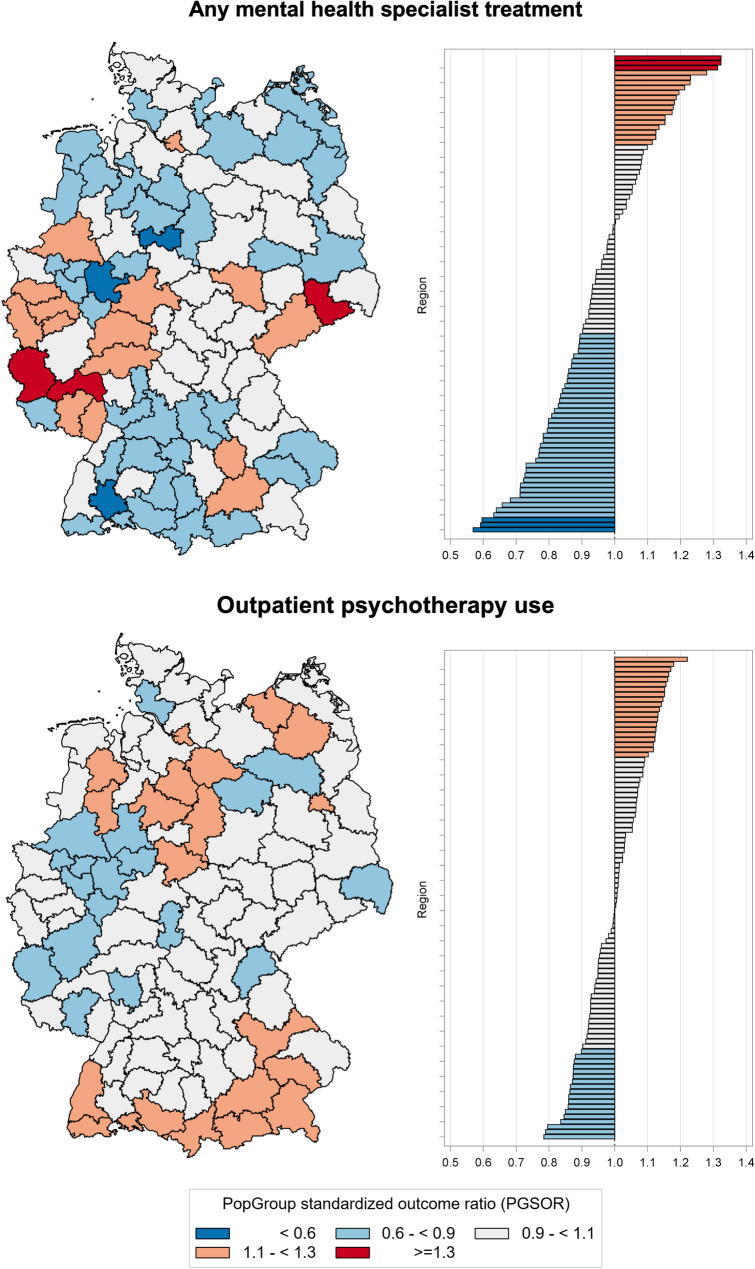
PopGroup standardized outcome ratios (PGSOR) for any outpatient mental health specialist contact and outpatient psychotherapy use. Note: Red shades represent regions with larger than average, grey shades regions with approximately average, and blue shades regions with below average proportions of any outpatient mental health specialist contact and outpatient psychotherapy service use

Regional variation was observed for all healthcare utilization indicators, with mean total healthcare costs per patient ranging from 4,676€ to 6,311€ per person, average days of sickness absence from 62.3 to 103.8 days, and proportions of any outpatient mental health specialist contact and outpatient psychotherapy use ranging from 10 to 25% and 14 to 22%, respectively. Figures 2 and 3 display PopGroup standardized outcome ratios (PGSOR), illustrating relative regional performance compared with the national average. Regional variation in healthcare costs was limited, with only seven regions deviating by more than ± 10% from the national mean, all located in southern Germany. In contrast, greater variation was observed for days of sickness absence (PGSOR range: 0.76–1.21), with above-average values predominantly found in northern and western regions.

PopGroup standardized regional variation was greatest for any outpatient mental health specialist contact (PGSOR: 0.56–1.32), with six regions – mainly in western Germany – deviating by more than ± 30% from the national average. In contrast, regional variation in outpatient psychotherapy use was moderate (PGSOR: 0.78–1.22).

Boxplots indicating the distribution of healthcare utilization by socioeconomic deprivation quintiles and level of urbanization can be accessed in Supplement C.

### Multilevel regression results

#### Socioeconomic deprivation

**Fig. 4:**
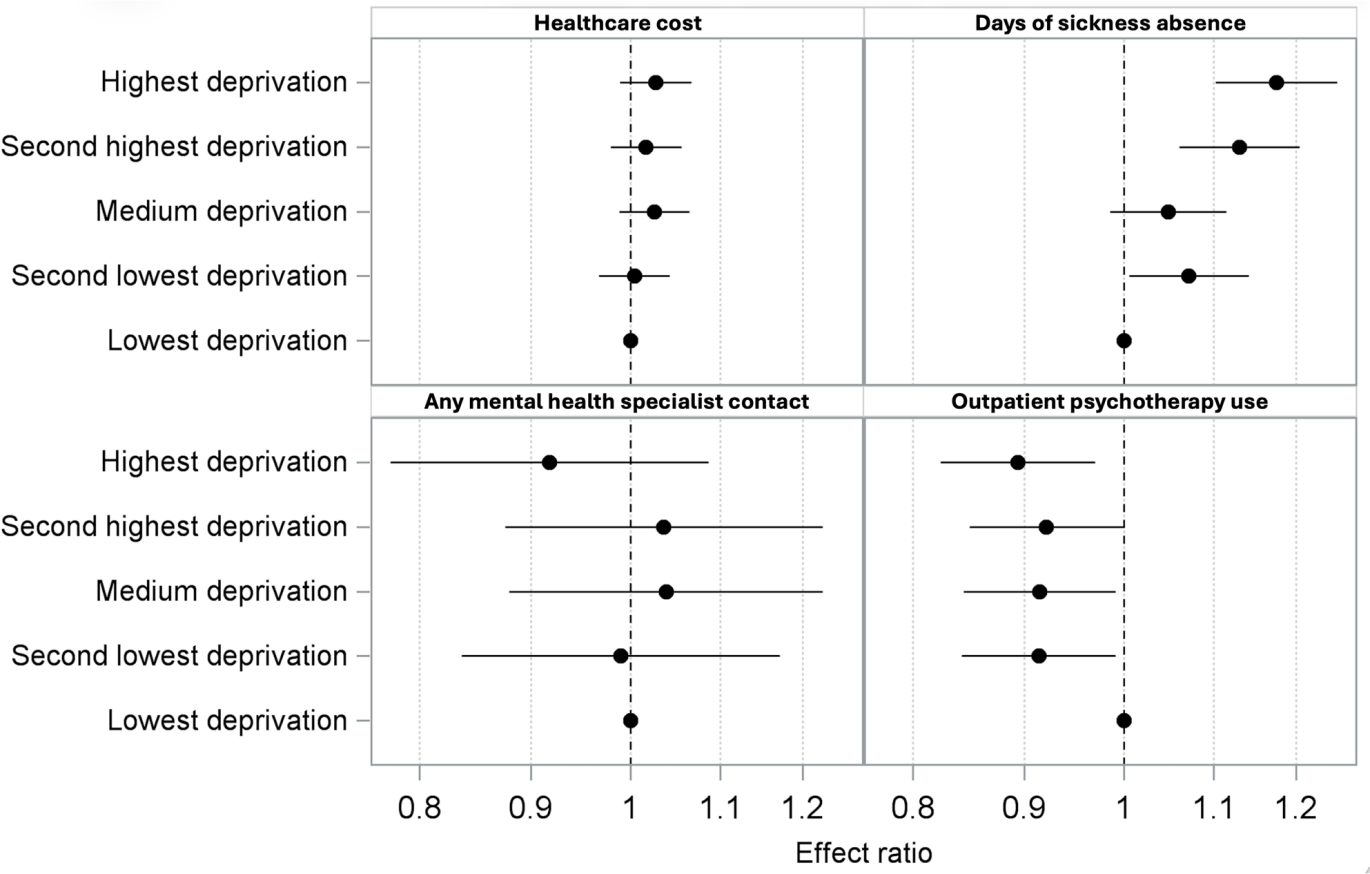
Effect ratios based on multilevel regression results presenting association between socioeconomic deprivation and depression-related healthcare utilization. Note: Points represent effect ratios (exp(β)) from multilevel regression model M2DEP adjusted for deprivation, age, sex and (Macro)PopGroup. Error bars indicate 95% confidence intervals. The lowest deprivation quintile is the reference category. Values above 1 indicate higher levels of the outcome compared to the reference, while values below 1 indicate lower levels. The vertical dashed line indicates no difference (effect ratio = 1)

Figure 4 presents effect ratios from the multilevel regression models assessing associations between regional socioeconomic deprivation and depression-related healthcare utilization, adjusted for age, sex, and PopGroups (M2DEP). A positive socioeconomic gradient was observed for days of sickness absence, with effect ratios increasing monotonically across deprivation quintiles, while no significant association was found for healthcare costs.

Any outpatient mental health specialist contact showed no significant association with deprivation, whereas outpatient psychotherapy use was inversely associated, indicating lower utilization in more deprived regions. Complete regression results for models M1DEP and M2DEP are provided in Supplement D. Effect ratios for the 19 (Macro)PopGroups are plotted in Supplement E.

Table 4 presents adjusted means and predicted probabilities on the natural scale by deprivation quintile. Adjusted healthcare costs showed only small differences across quintiles (2,178€ in the least vs. 2,236€ in the most deprived regions), whereas days of sickness absence increased more markedly from 34.1 to 40.1 days. In contrast, the adjusted probability of any outpatient mental health specialist contact declined slightly with increasing deprivation (approximately 14–15% in less deprived regions vs. 13.2% in the most deprived), with a similar pattern observed for outpatient psychotherapy use (17.9% vs. 16.3%).

In summary, higher regional deprivation was associated with greater sickness absence and slightly lower utilization of any outpatient mental health specialist contact and outpatient psychotherapy after adjustment for age, sex, and multimorbidity, indicating a mismatch between need and service use. Across all indicators, clear age and sex differences emerged: women had higher adjusted healthcare costs and were more likely to receive psychotherapeutic care, whereas men exhibited more days of sickness absence. Healthcare costs increased with age, while psychotherapeutic service use declined sharply, with the highest utilization among younger adults.

**Table 4:** Adjusted mean healthcare utilization by socioeconomic deprivation and patient characteristics

Variable	Healthcare cost in Euros			Days of sickness absence		
	Adj mean	LCL	UCL	Adj mean	LCL	UCL
Lowest deprivation	2,178	2,120	2,237	34.1	32.4	35.8
Second lowest	2,187	2,129	2,246	36.5	34.8	38.3
Medium deprivation	2,233	2,176	2,291	35.7	34.2	37.4
Second highest	2,214	2,155	2,273	38.5	36.7	40.4
Highest deprivation	2,236	2,177	2,297	40.1	38.2	42.1
Female	2,346	2,317	2,374	35.6	34.6	36.6
Male	2,081	2,054	2,107	38.4	37.2	39.6
Age 18–39	1,538	1,517	1,559	28.7	27.8	29.7
Age 40–59	1,918	1,894	1,942	36.3	35.3	37.3
Age 60–69	2,195	2,167	2,223	48.3	46.6	50.0
Age 70–79	2,741	2,705	2,777	.	.	.
Age > = 80	2,964	2,925	3,004	.	.	.
	*Estimates for 19 (Macro) PopGroups omitted for readability*			*Estimates for 19 (Macro) PopGroups omitted for readability*		
N	1,083,319			66,259		

**Variable**	**Any outpatient mental health specialist contact**			**Outpatient psychotherapy use**		
	Adj mean	LCL	UCL	Adj mean	LCL	UCL
Lowest deprivation	14.2%	12.8%	15.7%	17.9%	17.1%	18.8%
Second lowest	14.1%	12.7%	15.6%	16.6%	15.8%	17.4%
Medium deprivation	14.7%	13.3%	16.2%	16.6%	15.9%	17.4%
Second highest	14.6%	13.2%	16.2%	16.7%	15.9%	17.6%
Highest deprivation	13.2%	11.9%	14.6%	16.3%	15.5%	17.1%
Female	15.6%	14.9%	16.3%	18.1%	17.7%	18.5%
Male	12.8%	12.2%	13.4%	15.6%	15.3%	16.0%
Age 18–39	28.5%	27.4%	29.6%	21.0%	20.5%	21.4%
Age 40–59	23.2%	22.2%	24.2%	19.7%	19.3%	20.1%
Age 60–69	16.2%	15.5%	17.0%	16.1%	15.8%	16.5%
Age 70–79	8.4%	8.0%	8.8%	15.5%	15.1%	15.9%
Age > = 80	5.4%	5.1%	5.7%	12.9%	12.6%	13.3%
	*Estimates for 19 (Macro) PopGroups omitted for readability*			*Estimates for 19 (Macro) PopGroups omitted for readability*		
N	1,083,319			1,083,319		

Note: Adjusted means (adj mean), lower 95% confidence level (LCL) and upper 95% confidence level (UCL) from multilevel regression model M2DEP. Estimates for (Macro)PopGroups are omitted for readability. Sample sizes (N) refer to the number of individuals included in each model

#### Level of urbanization

**Fig. 5:**
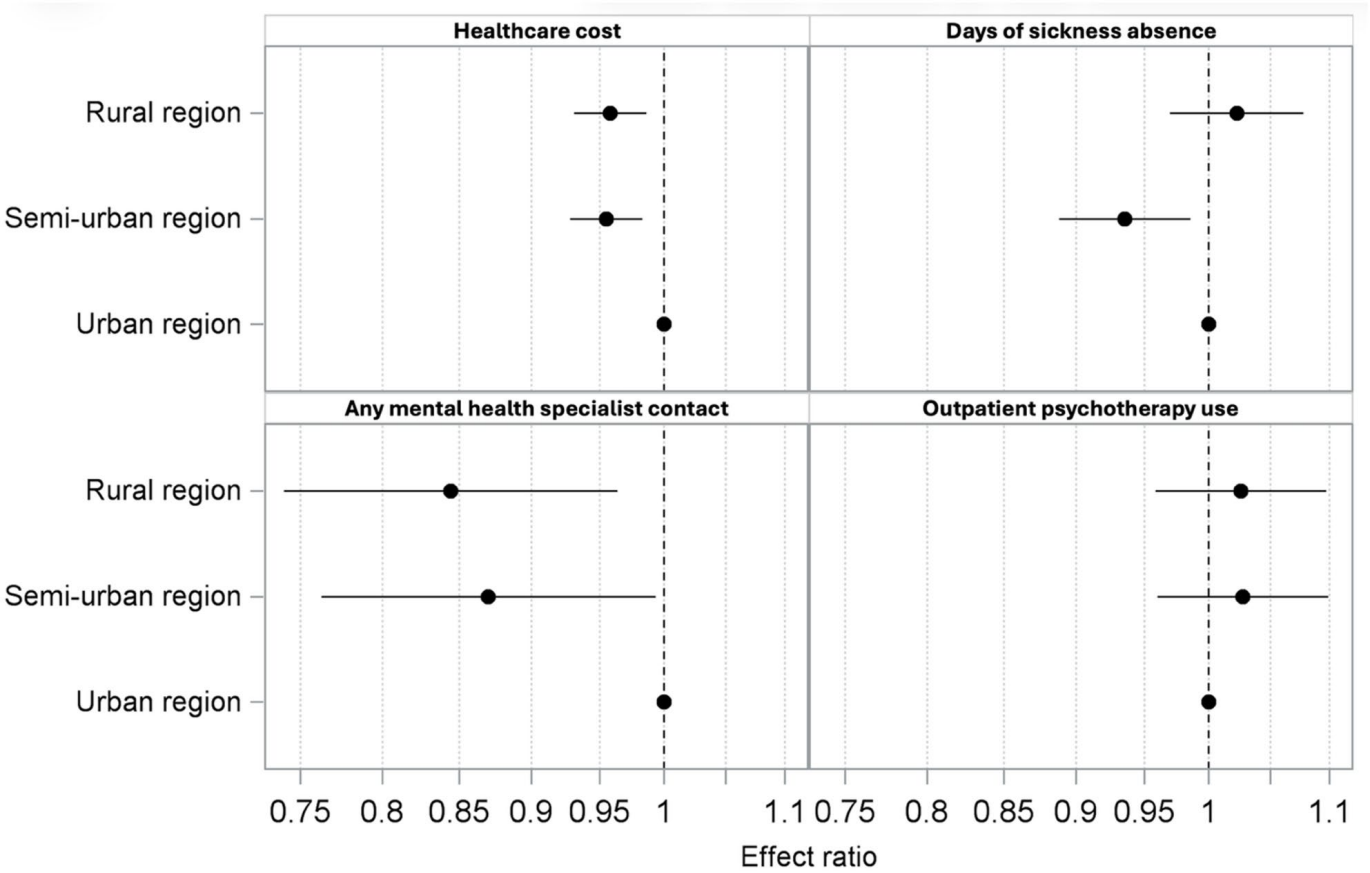
Effect ratios based on multilevel regression results presenting association between level of urbanization and depression-related healthcare utilization. Note: Points represent effect ratios (exp(β)) from multilevel regression model M2URB adjusted for urbanization, age, sex and (Macro)PopGroup. Error bars indicate 95% confidence intervals. Urban region is the reference category. Values above 1 indicate higher levels of the outcome compared to the reference, while values below 1 indicate lower levels. The vertical dashed line indicates no difference (effect ratio = 1)

Figure 5 displays effect ratios from multilevel regression models assessing associations between level of urbanization and depression-related healthcare utilization, adjusted for age, sex, and PopGroups. Associations with urbanization were generally weaker than those observed for socioeconomic deprivation. Healthcare costs were slightly lower in semi-urban and rural regions than in urban regions, while days of sickness absence were modestly higher in rural regions. Any outpatient mental health specialist contact was less likely in both semi-urban and rural regions compared with urban regions, whereas outpatient psychotherapy use did not differ significantly by urbanization level. Complete regression results for models M1URB and M2URB are provided in Supplement D.

Table 5 presents adjusted means and predicted probabilities by level of urbanization. Healthcare costs were highest in urban regions (2,284€) and slightly lower in semi-urban (2,181€) and rural regions (2,188€). Days of sickness absence were highest in rural regions (38.3 days) compared with urban (37.5 days) and semi-urban regions (35.1 days). Any outpatient mental health specialist contact was most frequent in urban regions (15.6%) and less common in semi-urban (13.9%) and rural regions (13.5%). In contrast, outpatient psychotherapy use varied only minimally across urbanization levels (16.6–16.9%).

Urbanization was associated with modest differences in costs and sickness absence, while any outpatient mental health specialist contact was more frequent in urban regions. Outpatient psychotherapy use showed little variation by urban–rural context. Again, clear age and sex differences emerged.

**Table 5:** Adjusted mean healthcare utilization by level of urbanization and patient characteristics

Variable	Healthcare cost in Euros				Days of sickness absence				
	Adj mean	LCL	UCL		Adj mean	LCL			UCL
Urban region	2,284	2,234	2,334		37.5	36.0			39.1
Semi-urban region	2,181	2,141	2,222		35.1	33.7			36.5
Rural region	2,188	2,147	2,229		38.3	36.8			40.0
Female	2,354	2,326	2,382		35.6	34.6			36.6
Male	2,088	2,063	2,114		38.4	37.1			39.6
Age 18–39	1,543	1,523	1,564		28.7	27.8			29.7
Age 40–59	1,925	1,902	1,948		36.3	35.3			37.4
Age 60–69	2,203	2,176	2,230		48.3	46.6			50.1
Age 70–79	2,751	2,716	2,786		.	.			.
Age > = 80	2,975	2,937	3,013		.	.			.
	*Estimates for 19 (Macro)PopGroups omitted for readability*				*Estimates for 19 (Macro)PopGroups omitted for readability*				
N	1,083,319				66,259				

**Variable**	**Any outpatient mental health specialist contact**				**Outpatient psychotherapy use**				
	Adj mean	LCL		UCL	Adj mean		LCL	UCL	
Urban region	15.6%	14.3%		17.0%	16.6%		15.9%	17.3%	
Semi-urban region	13.9%	12.9%		14.9%	16.9%		16.3%	17.6%	
Rural region	13.5%	12.6%		14.5%	16.9%		16.3%	17.5%	
Female	15.7%	15.1%		16.5%	18.1%		17.7%	18.5%	
Male	13.0%	12.4%		13.6%	15.6%		15.2%	16.0%	
Age 18–39	28.8%	27.7%		29.9%	20.9%		20.4%	21.4%	
Age 40–59	23.4%	22.5%		24.4%	19.7%		19.2%	20.1%	
Age 60–69	16.4%	15.7%		17.2%	16.1%		15.7%	16.5%	
Age 70–79	8.5%	8.1%		8.9%	15.5%		15.1%	15.9%	
Age > = 80	5.4%	5.2%		5.7%	12.9%		12.6%	13.2%	
	*Estimates for 19 (Macro)PopGroups omitted for readability*				*Estimates for 19 (Macro)PopGroups omitted for readability*				
N	1,083,319				1,083,319				

Note: Adjusted means (adj mean), lower 95% confidence levels (LCL) and upper 95% confidence levels (UCL) from multilevel regression model M2URB. Estimates for (Macro)PopGroups are omitted for readability. Sample sizes (N) refer to the number of individuals included in each model

#### Results of proportional variance component analysis

Table 6 presents the results of the proportional variance component analysis. Across all healthcare utilization indicators, regional clustering was present but small to modest, with ICCs ranging from approximately 0.3% to 0.4% for the continuous indicators and from 0.5% to 2.0% for the binary indicators in the null models (M0).

**Table 6:** Proportional variance components analysis results

Indicator	Model	ICC	PCV	Indicator	Model	ICC	PCV
Healthcare cost	M0	0.3%	0.0%	Days of sickness absence	M0	0.4%	0.0%
	M1DEP	0.3%	10.2%		M1DEP	0.3%	23.9%
	M1URB	0.3%	2.9%		M1URB	0.4%	9.6%
	M2DEP	0.2%	46.9%		M2DEP	0.3%	40.3%
	M2URB	0.2%	53.1%		M2URB	0.3%	23.6%
Any outpatient mental health specialist contact	M0	1.8%	0.0%	Outpatient psychotherapy use	M0	0.5%	0.0%
	M1DEP	1.8%	-0.7%		M1DEP	0.4%	4.4%
	M1URB	1.6%	7.5%		M1URB	0.5%	-1.8%
	M2DEP	2.0%	-16.9%		M2DEP	0.5%	-0.5%
	M2URB	1.9%	-9.8%		M2URB	0.5%	-7.2%

Note: Intra-class correlation coefficients (ICC) and proportional change in variance (PCV) are reported for each healthcare utilization indicator and model. Positive PCV values indicate a reduction in regional variance relative to the null model, whereas negative PCV values indicate an increase in regional variance after adjustment. M0 = null model, M1DEP = deprivation only, M1URB = urbanization only, M2DEP = deprivation, age, sex, morbidity, M2URB = urbanization, age, sex, morbidity

For healthcare costs and sickness absence, regional deprivation (M1DEP) explained 10.2% and 23.9% of the small observed between-region variance. Adding patient-level characteristics (M2DEP) substantially increased PCVs to 46.9% and 40.3%. Urbanization only (M1URB) explained less variance (2.9% and 9.6%) but again adding patient characteristics (M2URB) substantially increased PCVs to 53.1% and 23.6%.

For any outpatient mental health specialist contact, between-region variance was highest (ICC ≈ 1.8%) and remained largely unexplained by the covariates. Instead, between-region variance increased (indicated by negative PCVs) after adjusting for deprivation only (-0.7%) and additional patient factors (-16.9%). A similar pattern was observed for outpatient psychotherapy use, with negative PCVs of -0.5% to -7.2% for the fully adjusted models. This indicates that regional heterogeneity in psychotherapeutic care remains largely unexplained by the included covariates. A detailed visualization of region-level random effects from the fully adjusted models is provided in Supplement F.

## Discussion

This study examined regional variation in multiple indicators of depression-related healthcare resource use in Germany using nationwide claims data, multilevel modeling, and the PopGrouper as a population classification system to account for multimorbidity. Several key findings emerge. First, observed regional variation in depression-related healthcare utilization was relatively small and most pronounced for the likelihood of any outpatient mental health specialist contact. Second, higher regional socioeconomic deprivation was associated with a greater number of days of sickness absence and a slightly lower likelihood of receiving outpatient psychotherapy, even after adjustment for age, sex, and multimorbidity. Third, the likelihood of having any outpatient mental health specialist contact was highest in urban regions whereas outpatient psychotherapy use appeared largely independent of urban–rural context after adjustment. Finally, the overall small regional clustering remained largely unexplained for any outpatient mental health specialist contact and outpatient psychotherapy use, indicating persistent unexplained regional heterogeneity in access to mental health services.

The observed positive socioeconomic gradient in sickness absence aligns well with prior evidence from Germany and other countries that has consistently demonstrated higher depression prevalence, greater symptom severity, and longer sickness absence durations in deprived areas [3, 5, 7, 8, 16]. Our findings extend this literature by showing that these patterns persist even after adjusting for detailed multimorbidity profiles, suggesting that regional deprivation captures contextual factors beyond individual health status alone. These may include adverse working conditions, psychosocial stressors, reduced social capital, and limited access to supportive resources, all of which are known social determinants of mental health [18]. The absolute differences in days of sickness absence between the least and most deprived regions were substantial, exceeding six days on average after adjustment. From a health system perspective, this highlights the broader economic implications of regional inequalities in mental health, extending beyond direct healthcare expenditures to productivity losses and social insurance costs.

Even after adjusting for multimorbidity and demographic characteristics, we observed slightly lower probabilities of receiving outpatient psychotherapy in more deprived regions. This suggests that contextual barriers—such as longer waiting times, lower provider density, reduced health literacy, or higher opportunity costs—may disproportionately affect individuals in these areas, despite the absence of direct financial barriers in the German healthcare system. This mismatch between need and utilization mirrors findings from survey-based and claims data studies in Germany, which have repeatedly documented substantial treatment gaps in depression care [9–12]. Engels et al. [12], for example, showed that only 24–40% of individuals received guideline-recommended psychotherapy or pharmacotherapy within the first year after diagnosis.

Differences by level of urbanization were generally smaller than those observed for socioeconomic deprivation, but clear patterns emerged for healthcare costs and any outpatient mental health specialist contact. Individuals living in urban regions had higher healthcare costs and were more likely to have any outpatient mental health specialist contact than those in semi-urban or rural regions. This finding is in line with previous studies demonstrating higher utilization of psychotherapeutic and psychiatric services in regions with higher provider density and better supply structures [9]. It also reflects well-documented urban–rural disparities in specialist availability in Germany.

Interestingly, outpatient psychotherapy use did not differ meaningfully by urbanization level after adjustment. This may indicate that once individuals enter psychotherapeutic care, the likelihood of receiving guideline-concordant services is relatively similar across regions. Alternatively, it may reflect limitations of claims-based measures in capturing treatment intensity and quality. Nonetheless, the divergence between any psychotherapist contact and outpatient psychotherapy use underscores the importance of distinguishing between access to providers and receipt of evidence-based care.

The proportional variance component analysis showed that the small observed between-region variance in healthcare costs and sickness absence could by large parts be explained by regional socioeconomic deprivation, age, sex and morbidity and to a lesser extent by urbanization. In contrast, these contextual factors explained little of the regional heterogeneity in any outpatient mental health specialist contacts and outpatient psychotherapy use. For any outpatient mental health specialist contact, regional variance even increased after adjustment, suggesting that individual-level controls may unmask underlying structural differences in service provision rather than attenuate them. These results suggest that while regional socioeconomic context is linked to sickness absence, access to psychotherapeutic care is partly explained by urbanization but rather shaped by additional, more localized factors—such as provider supply, referral practices, and organizational characteristics of mental health services—that were not captured in the present analysis.

## Strengths and limitations

A major strength of this study is the use of large-scale, nationwide claims data covering over one million individuals with diagnosed depression. This allowed for detailed regional analyses and avoided biases associated with self-reported diagnoses and service use. Another key strength is the application of the PopGrouper for morbidity adjustment. By accounting for multimorbidity in a comprehensive way, the PopGrouper enables more meaningful regional comparisons than age–sex standardization alone and reduces the risk that observed differences merely reflect variations in population health status. The multilevel modeling approach further strengthens the analysis by explicitly accounting for clustering at the regional level and allowing quantification of explained and unexplained regional variance.

Several limitations should be acknowledged. First, the analysis relies on data from a single sickness fund, covering approximately 10% of the German population. Although BARMER has nationwide coverage, differences in insured populations across sickness funds may limit generalizability. Second, claims data capture only administratively coded depression diagnoses. Underdiagnosis remains an important limitation of administrative data, and treated depression that is not coded as such is not included. At the same time, overdiagnosis or diagnostic upcoding may also occur, for example to justify treatment, particularly in regions with higher provider density. Moreover, claims data do not include standardized clinical assessments of depression severity. As a result, the observed prevalence and patterns of care reflect recorded diagnoses rather than the underlying epidemiology of depression. This may also contribute to observed regional variation, as coding practices and diagnostic thresholds may differ across regions. Third, our measures of psychotherapeutic care capture contact and service use but not treatment adequacy, duration, or patient preferences. Fourth, socioeconomic deprivation was measured at the regional level and cannot substitute individual-level socioeconomic status, which may operate differently. Finally, the cross-sectional nature of the analysis limits causal inference.

## Implications for practice and research

From a policy and practice perspective, the findings primarily highlight the strong association between regional socioeconomic deprivation and days of sickness absence among individuals with depression. Even after adjustment for age, sex, and multimorbidity, individuals living in more deprived regions exhibited substantially longer periods of certified sickness absence. This suggests that contextual socioeconomic factors contribute not only to mental health burden but also to prolonged work disability.

For research, our results underscore the value of combining detailed morbidity adjustment with multilevel approaches to better understand regional inequalities in mental healthcare. Future studies should integrate supply-side indicators, such as provider density and waiting times, as well as individual-level socioeconomic data, to further disentangle mechanisms underlying regional variation. Extending the analysis to data from all German sickness funds recently available through the national Health Data Lab, would allow more comprehensive assessment of regional patterns and improve generalizability.

## Supplementary Information

Below is the link to the electronic supplementary material.

Supplementary Material 1

Supplementary Material 2

Supplementary Material 3

Supplementary Material 4

Supplementary Material 5

Supplementary Material 6

## Data Availability

The data utilized in this current study are health insurance claims data provided by the German statutory health insurance fund BARMER. Due to legal and privacy restrictions, these data are not publicly available. Access to the data is subject to approval by the BARMER and may require additional agreements regarding data protection and confidentiality.
