# Supplementary material for: Regional variation in healthcare utilization among patients with depression in Germany: a multilevel analysis with PopGrouper-based multimorbidity adjustment
Source: Res Health Serv Reg. 2026 Jun 9;5:8. doi: 10.1007/s43999-026-00092-6 (PMC13250020; doi:10.1007/s43999-026-00092-6)
Supplement: Supplementary file 1 — Supplementary Material 1 [file 43999_2026_92_MOESM1_ESM.pdf]

Supplement A: Diagnosis selection criteria for study population

The study population was selected based on consolidated disease groups (ZKGs) from the PopGrouper version 1.0 [1]. ZKGs build on diagnosis groups (DxGs) used in the German health insurance system to adjust funding received by the health insurers for morbidity [2]. The DxGs are based on ICD-10 codes from the International Statistical Classification of Diseases and Related Health Problems (10th Revision) and additional selection criteria as listed below.

Persons having at least one of the following ZKGs were included:

ZKG	ZKG title	DxG	DxG title	Selection criteria for DxG classifcation[1]				
				Age	ICD-10 codes	Inpatient diagnosis required[2]	Medication required[3]	Minimum treatment length
Z1104	Severe depression	838	Recurrent major depression	-	F33.2-3	no	yes	>=10 days
		839	Severe depression without recurrence	-	F33.2-3	no	yes	>=10 days
Z1105	Mild to moderate depression	271	Recurrent depressive disorder (Age <55)	<55	F33.0; F33.1; F33.4	no	no	no
		272	Recurrent depressive disorder (Age >54)	>54	F33.0; F33.1; F33.4	no	no	no
		273	Mild and moderate depressive episodes (Age <50)	<50	F32.0-1	no	no	no
		274	Mild and moderate depressive episodes (Age >49)	>49	F32.0-1	no	no	no
		476	Other or unspecified affective or neurotic disorders, other neurotic disorders	-	F34.8-9, F38.0-1, F38.8, F39, F48.0, F48.9, F99	no	no	no
		817	Other or unspecified recurrent depressive disorders	-	F33.8-9	no	no	no
		818	Other depressive episodes	-	F32.8-9	no	no	no
		844	Dysthymia	-	F34.1	no	no	no
		1241	Other persistent affective disorders	-	F41.2	no	no	no

Note: 1 based on [2]; 2 if "no", confirmed outpatient diagnosis in at least 2 quarters of the year is sufficient; 3 if "yes", at least 1 simultanuous medication prescription required.

[1] Braun A, Grobe TG, Tsatsaronis C, et al. [Grouping Algorithm of the PopGrouper Version 1.0]. Working papers in health policy & management. Available via the institutional repository of Technische Universität Berlin; 2025. https://doi.org/10.14279/depositonce-23953

[2] German Federal Office for Social Security. Risikostrukturausgleich: Festlegungen. 2025. Accessed January 16, 2025. https://www.bundesamtsozialesicherung.de/de/themen/risikostrukturausgleich/festlegungen/
