# Supplementary material for: Regional variation in healthcare utilization among patients with depression in Germany: a multilevel analysis with PopGrouper-based multimorbidity adjustment
Source: Res Health Serv Reg. 2026 Jun 9;5:8. doi: 10.1007/s43999-026-00092-6 (PMC13250020; doi:10.1007/s43999-026-00092-6)
Supplement: Supplementary file 2 — Supplementary Material 2 [file 43999_2026_92_MOESM2_ESM.pdf]

Supplement B: Model fit statistics

Table B-1: Model Fit Statistics for Multilevel Regression Models by healthcare utilization indicator

Indicator	Model	Neg2LL	AIC	ΔAIC	BIC	ΔBIC
Healthcare cost	M2DEP	3528034.59	3528038.59	20	3528043.72	20
	M2URB	3528014.30	3528018.30	0	3528023.42	0
	M3ADD	3528035.37	3528039.37	21	3528044.50	21
	M3INT	3528072.00	3528076.00	58	3528081.12	58
Days of sickness absence	M2DEP	235396.01	235400.01	0	235405.14	0
	M2URB	235401.32	235405.32	6	235410.45	6
	M3ADD	235395.52	235399.52	0	235404.65	0
	M3INT	235419.50	235423.50	24	235428.62	24
Any outpatient mental health specialist contact	M2DEP	969690.58	969748.58	8	969822.95	13
	M2URB	969686.66	969740.66	0	969809.90	0
	M3ADD	969684.60	969746.60	6	969826.10	16
	M3INT	969674.20	969752.20	12	969852.21	42
Outpatient psychotherapy use	M2DEP	1002173.43	1002231.43	0	1002305.79	1
	M2URB	1002181.47	1002235.47	4	1002304.71	0
	M3ADD	1002171.31	1002233.31	2	1002312.80	8
	M3INT	1002166.54	1002244.54	13	1002344.55	40

Note: All models adjusted for age, sex, (Macro)PopGroup. Additionally, M2DEP adjusts for deprivation, M2URB for urbanization, M3ADD for deprivation and urbanization, and M3INT for deprivation and urbanization and its interaction. Models were estimated using the same method and analytic sample per outcome. ΔAIC and ΔBIC refer to the difference from the best-fitting model (lowest value) within each outcome.
