# Supplementary material for: Regional variation in healthcare utilization among patients with depression in Germany: a multilevel analysis with PopGrouper-based multimorbidity adjustment
Source: Res Health Serv Reg. 2026 Jun 9;5:8. doi: 10.1007/s43999-026-00092-6 (PMC13250020; doi:10.1007/s43999-026-00092-6)
Supplement: Supplementary file 3 — Supplementary Material 3 [file 43999_2026_92_MOESM3_ESM.pdf]

## Supplement C: Boxplots of healthcare utilization indicators by socioeconomic deprivation quintiles and level of urbanization

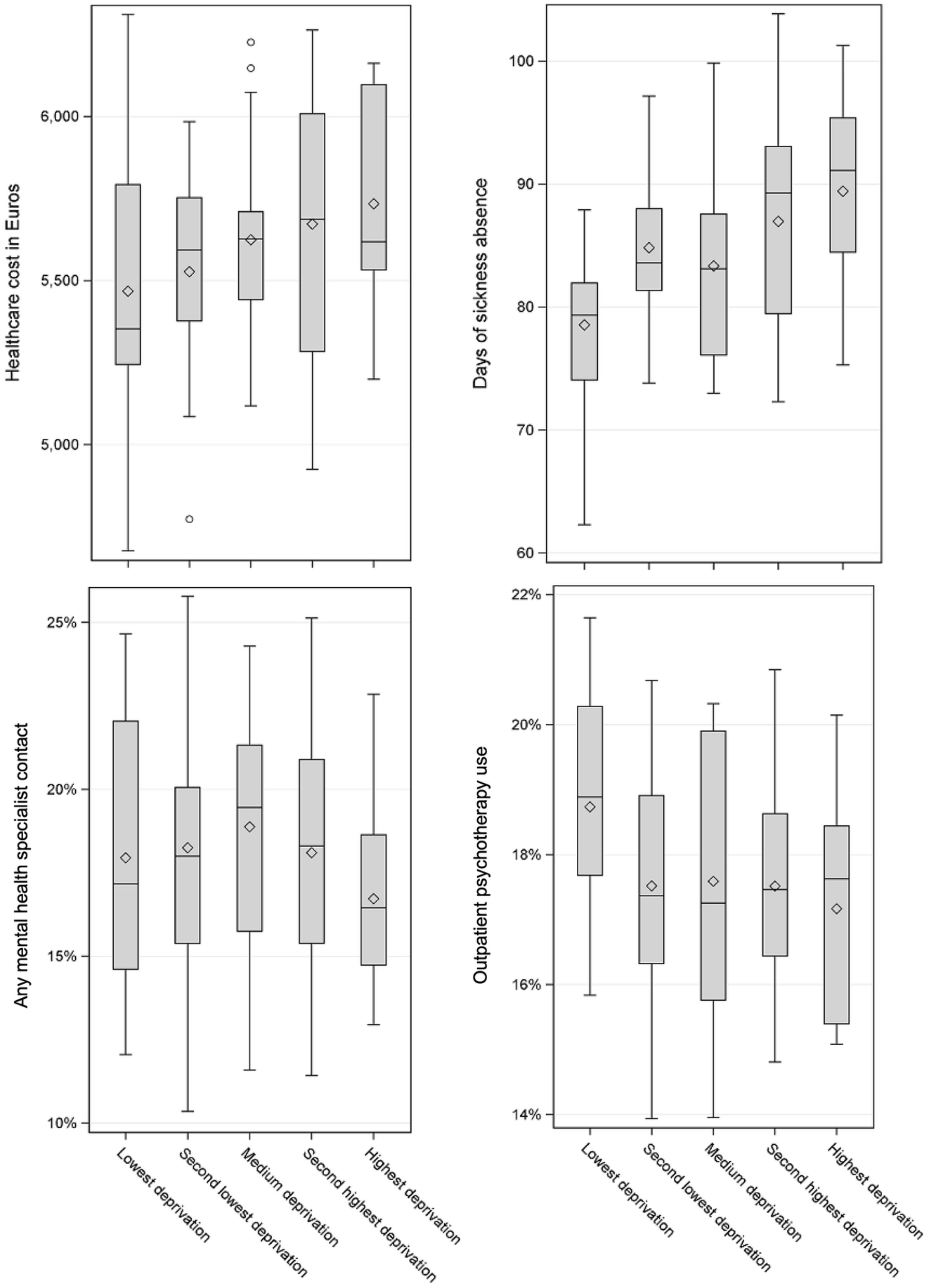


Figure C-1: Boxplots of regional healthcare utilization distribution by socioeconomic deprivation quintiles
Note: Plotted are means across the 96 regions. Diamonds indicate the mean, horizontal lines within the box the median, the box the interquartile range, and the whiskers the range excluding outliers. Healthcare costs are presented in average Euros per person.

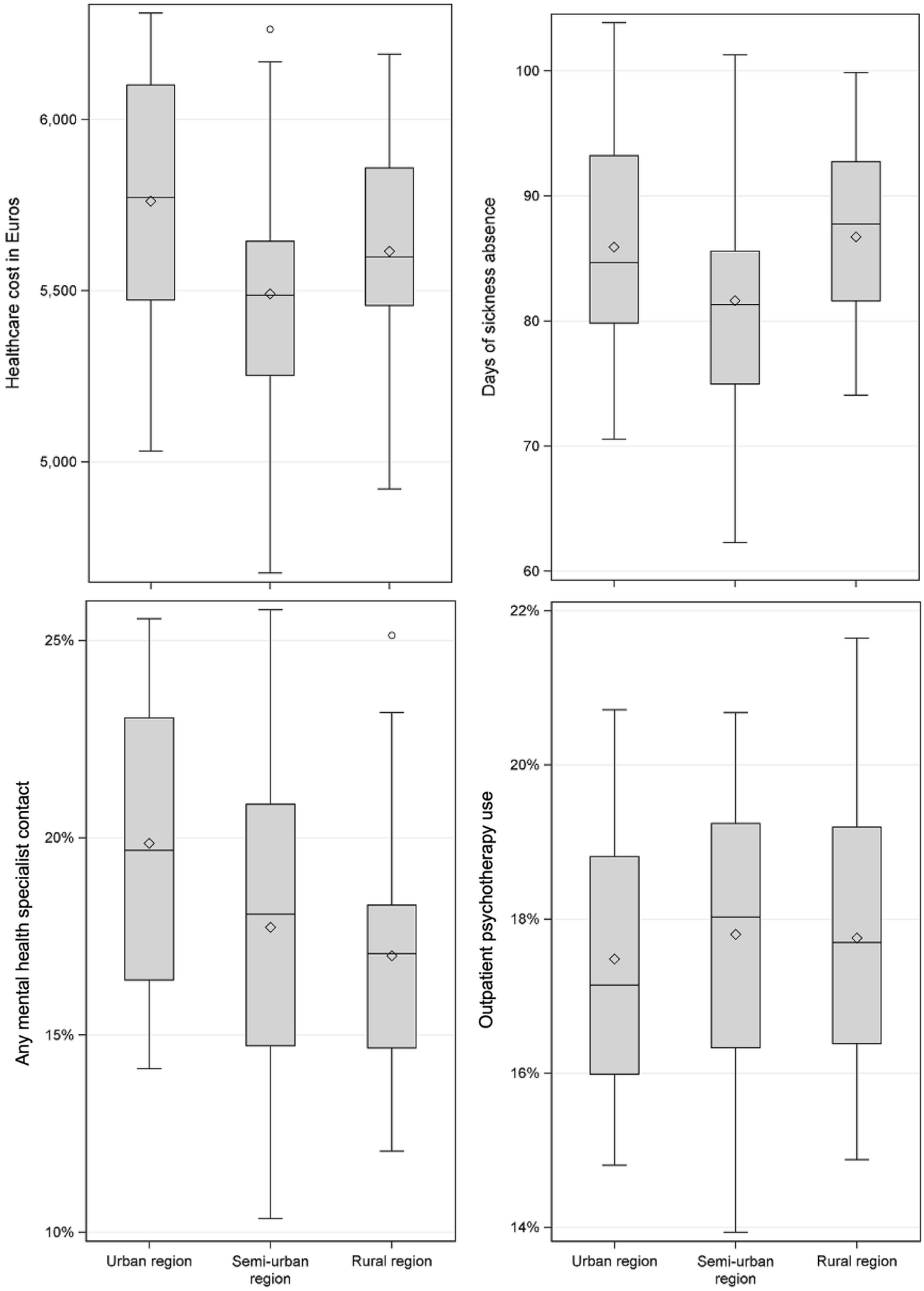


Figure C-2: Boxplots of regional healthcare utilization distribution by level of urbanization
Note: Plotted are mean outcomes across the 96 regions. Diamonds indicate the mean, horizontal lines within the box the median, the box the interquartile range, and the whiskers the range excluding outliers. Healthcare costs are presented in average Euros per person.
