# Supplementary material for: Regional variation in healthcare utilization among patients with depression in Germany: a multilevel analysis with PopGrouper-based multimorbidity adjustment
Source: Res Health Serv Reg. 2026 Jun 9;5:8. doi: 10.1007/s43999-026-00092-6 (PMC13250020; doi:10.1007/s43999-026-00092-6)
Supplement: Supplementary file 4 — Supplementary Material 4 [file 43999_2026_92_MOESM4_ESM.pdf]

Supplement D: Fixed effects from multilevel regression models

Table D-1: Fixed effects results from models M1DEP and M2DEP with indicator "Healthcare cost"

	Healthcare cost					
	M1DEP			M2DEP		
**Variable**	Est	SE	p-value	Est	SE	p-value
Intercept	7.570	0.014	0.000	6.714	0.014	0.000
Lowest deprivation	0.000	.	.	0.000	.	.
Second lowest	0.021	0.022	0.357	0.004	0.019	0.822
Medium deprivation	0.047	0.022	0.036	0.025	0.019	0.180
Second highest	0.050	0.022	0.028	0.016	0.019	0.387
Highest deprivation	0.061	0.022	0.008	0.026	0.019	0.165
Female	0.000	.	.	0.000	.	.
Male	-0.075	0.003	0.000	-0.120	0.003	0.000
Age 18-39	-0.310	0.004	0.000	-0.221	0.004	0.000
Age 40-59	0.000	.	.	0.000	.	.
Age 60-69	0.248	0.003	0.000	0.135	0.003	0.000
Age 70-79	0.585	0.004	0.000	0.357	0.004	0.000
Age >=80	0.772	0.004	0.000	0.435	0.004	0.000
P07060BB	.	.	.	0.000	.	.
P05106BB	.	.	.	0.717	0.008	0.000
P06023BB	.	.	.	1.062	0.006	0.000
P06028BB	.	.	.	1.380	0.012	0.000
P06034BB	.	.	.	1.232	0.009	0.000
P06045BB	.	.	.	1.279	0.010	0.000
P06063BB	.	.	.	0.889	0.010	0.000
P06067BB	.	.	.	0.549	0.012	0.000
P06087BB	.	.	.	0.422	0.012	0.000
P07010BB	.	.	.	0.932	0.011	0.000
P07025BZ	.	.	.	0.528	0.009	0.000
P07042BB	.	.	.	0.493	0.010	0.000
P07050BB	.	.	.	0.289	0.012	0.000
P07052ZB	.	.	.	0.321	0.009	0.000
P03	.	.	.	1.949	0.007	0.000
P04	.	.	.	1.779	0.009	0.000
P05	.	.	.	1.491	0.005	0.000
P06	.	.	.	0.952	0.005	0.000
P07	.	.	.	0.658	0.005	0.000
N		1,083,319			1,083,319	

Note: Fixed effects estimates (Est), standard errors (SE) and p-values based on multilevel regression models with M1DEP including socioeconomic deprivation quintiles, age groups, and sex as covariate and M2DEP additionally including 19 (Macro) PopGroups. Covariates with Est=0.000 are reference categories.

Table D-2: Fixed effects results from models M1DEP and M2DEP with indicator “Days of sickness absence”

	**Days of sickness absence**					
	**M1DEP**			**M2DEP**		
**Variable**	Est	SE	p-value	Est	SE	p-value
Intercept	3.491	0.025	0.000	3.355	0.027	0.000
Lowest deprivation	0.000	.	.	0.000	.	.
Second lowest	0.068	0.033	0.045	0.069	0.032	0.033
Medium deprivation	0.044	0.033	0.176	0.047	0.031	0.131
Second highest	0.106	0.034	0.002	0.122	0.032	0.000
Highest deprivation	0.150	0.034	0.000	0.161	0.032	0.000
Female	0.000	.	.	0.000	.	.
Male	0.094	0.012	0.000	0.076	0.012	0.000
Age 18-39	-0.237	0.013	0.000	-0.234	0.013	0.000
Age 40-59	0.000	.	.	0.000	.	.
Age 60-69	0.285	0.015	0.000	0.286	0.015	0.000
Age 70-79	.	.	.	.	.	.
Age >=80	.	.	.	.	.	.
P07060BB	.	.	.	0.000	.	.
P05106BB	.	.	.	0.047	0.027	0.080
P06023BB	.	.	.	0.573	0.020	0.000
P06028BB	.	.	.	0.056	0.108	0.603
P06034BB	.	.	.	0.141	0.047	0.003
P06045BB	.	.	.	0.146	0.058	0.011
P06063BB	.	.	.	0.192	0.056	0.001
P06067BB	.	.	.	-0.026	0.042	0.538
P06087BB	.	.	.	-0.079	0.043	0.066
P07010BB	.	.	.	0.048	0.055	0.381
P07025BZ	.	.	.	0.058	0.046	0.211
P07042BB	.	.	.	0.112	0.044	0.011
P07050BB	.	.	.	0.273	0.038	0.000
P07052ZB	.	.	.	0.019	0.035	0.581
P03	.	.	.	0.365	0.052	0.000
P04	.	.	.	0.146	0.067	0.029
P05	.	.	.	0.091	0.023	0.000
P06	.	.	.	0.046	0.019	0.015
P07	.	.	.	0.045	0.021	0.029
N		66,259		66,259		

Note: Fixed effects estimates (Est), standard errors (SE) and p-values based on multilevel regression models with M1DEP including socioeconomic deprivation quintiles, age groups, and sex as covariate and M2DEP additionally including 19 (Macro) PopGroups. Covariates with Est=0.000 are reference categories.

Table D-3: Fixed effects results from models M1DEP and M2DEP with indicator “Any mental health specialist contact”

	Any mental health specialist contact					
	**M1DEP**			**M2DEP**		
**Variable**	Est	SE	p-value	Est	SE	p-value
Intercept	-1.037	0.060	0.000	-1.322	0.061	0.000
Lowest deprivation	0.000	.	.	0.000	.	.
Second lowest	-0.007	0.084	0.934	-0.010	0.086	0.904
Medium deprivation	0.049	0.083	0.555	0.038	0.085	0.657
Second highest	0.036	0.084	0.670	0.035	0.086	0.681
Highest deprivation	-0.083	0.084	0.325	-0.086	0.086	0.316
Female	0.000	.	.	0.000	.	.
Male	-0.213	0.006	0.000	-0.228	0.006	0.000
Age 18-39	0.292	0.007	0.000	0.278	0.007	0.000
Age 40-59	0.000	.	.	0.000	.	.
Age 60-69	-0.466	0.006	0.000	-0.443	0.007	0.000
Age 70-79	-1.236	0.009	0.000	-1.192	0.009	0.000
Age >=80	-1.705	0.010	0.000	-1.672	0.011	0.000
P07060BB	.	.	.	0.000	.	.
P05106BB	.	.	.	0.233	0.016	0.000
P06023BB	.	.	.	1.241	0.011	0.000
P06028BB	.	.	.	0.321	0.030	0.000
P06034BB	.	.	.	0.391	0.018	0.000
P06045BB	.	.	.	-0.180	0.025	0.000
P06063BB	.	.	.	-0.086	0.025	0.001
P06067BB	.	.	.	0.923	0.021	0.000
P06087BB	.	.	.	-0.012	0.024	0.639
P07010BB	.	.	.	-0.062	0.025	0.014
P07025BZ	.	.	.	-0.041	0.021	0.055
P07042BB	.	.	.	0.107	0.022	0.000
P07050BB	.	.	.	0.486	0.022	0.000
P07052ZB	.	.	.	0.535	0.017	0.000
P03	.	.	.	0.090	0.016	0.000
P04	.	.	.	0.102	0.022	0.000
P05	.	.	.	0.229	0.010	0.000
P06	.	.	.	0.278	0.010	0.000
P07	.	.	.	0.057	0.010	0.000
N	1,083,319			1,083,319		

Note: Fixed effects estimates (Est), standard errors (SE) and p-values based on multilevel regression models with M1DEP including socioeconomic deprivation quintiles, age groups, and sex as covariate and M2DEP additionally including 19 (Macro) PopGroups. Covariates with Est=0.000 are reference categories.

Table D-4: Fixed effects results from models M1DEP and M2DEP with indicator "Outpatient psychotherapy use"

	Outpatient psychotherapy use					
	**M1DEP**			**M2DEP**		
**Variable**	Est	SE	p-value	Est	SE	p-value
Intercept	-1.248	0.029	0.000	-1.390	0.031	0.000
Lowest deprivation	0.000	.	.	0.000	.	.
Second lowest	-0.089	0.041	0.031	-0.090	0.041	0.029
Medium deprivation	-0.086	0.041	0.034	-0.089	0.041	0.029
Second highest	-0.080	0.041	0.052	-0.082	0.042	0.047
Highest deprivation	-0.110	0.041	0.008	-0.112	0.042	0.007
Female	0.000	.	.	0.000	.	.
Male	-0.177	0.006	0.000	-0.175	0.006	0.000
Age 18-39	0.076	0.008	0.000	0.078	0.008	0.000
Age 40-59	0.000	.	.	0.000	.	.
Age 60-69	-0.241	0.007	0.000	-0.243	0.007	0.000
Age 70-79	-0.287	0.008	0.000	-0.289	0.008	0.000
Age >=80	-0.496	0.008	0.000	-0.503	0.008	0.000
P07060BB	.	.	.	0.000	.	.
P05106BB	.	.	.	0.272	0.016	0.000
P06023BB	.	.	.	0.332	0.012	0.000
P06028BB	.	.	.	0.141	0.028	0.000
P06034BB	.	.	.	0.293	0.018	0.000
P06045BB	.	.	.	0.053	0.021	0.014
P06063BB	.	.	.	0.108	0.022	0.000
P06067BB	.	.	.	0.139	0.025	0.000
P06087BB	.	.	.	0.128	0.025	0.000
P07010BB	.	.	.	0.128	0.023	0.000
P07025BZ	.	.	.	0.088	0.020	0.000
P07042BB	.	.	.	0.110	0.022	0.000
P07050BB	.	.	.	0.068	0.025	0.007
P07052ZB	.	.	.	0.114	0.019	0.000
P03	.	.	.	0.095	0.015	0.000
P04	.	.	.	0.318	0.019	0.000
P05	.	.	.	0.149	0.011	0.000
P06	.	.	.	0.161	0.010	0.000
P07	.	.	.	0.100	0.011	0.000
N		1,083,319			1,083,319	

Note: Fixed effects estimates (Est), standard errors (SE) and p-values based on multilevel regression models with M1DEP including socioeconomic deprivation quintiles, age groups, and sex as covariate and M2DEP additionally including 19 (Macro) PopGroups. Covariates with Est=0.000 are reference categories.

Table D-5: Fixed effects results from models M1URB and M2URB with indicator “Healthcare cost”

	**Healthcare cost**					
	**M1URB**			**M2URB**		
**Variable**	Est	SE	p-value	Est	SE	p-value
Intercept	7.570	0.014	0.000	6.762	0.012	0.000
Urban region	0.000	.	.	0.000	.	.
Semi-urban region	-0.051	0.018	0.005	-0.046	0.014	0.002
Rural region	-0.039	0.018	0.030	-0.043	0.014	0.004
Female	0.000	.	.	0.000	.	.
Male	-0.075	0.003	0.000	-0.120	0.003	0.000
Age 18-39	-0.310	0.004	0.000	-0.221	0.004	0.000
Age 40-59	0.000	.	.	0.000	.	.
Age 60-69	0.248	0.003	0.000	0.135	0.003	0.000
Age 70-79	0.585	0.004	0.000	0.357	0.004	0.000
Age >=80	0.772	0.004	0.000	0.435	0.004	0.000
P07060BB	.	.	.	0.000	.	.
P05106BB	.	.	.	0.717	0.008	0.000
P06023BB	.	.	.	1.062	0.006	0.000
P06028BB	.	.	.	1.380	0.012	0.000
P06034BB	.	.	.	1.232	0.009	0.000
P06045BB	.	.	.	1.279	0.010	0.000
P06063BB	.	.	.	0.889	0.010	0.000
P06067BB	.	.	.	0.549	0.012	0.000
P06087BB	.	.	.	0.422	0.012	0.000
P07010BB	.	.	.	0.932	0.011	0.000
P07025BZ	.	.	.	0.528	0.009	0.000
P07042BB	.	.	.	0.493	0.010	0.000
P07050BB	.	.	.	0.289	0.012	0.000
P07052ZB	.	.	.	0.321	0.009	0.000
P03	.	.	.	1.949	0.007	0.000
P04	.	.	.	1.779	0.009	0.000
P05	.	.	.	1.491	0.005	0.000
P06	.	.	.	0.952	0.005	0.000
P07	.	.	.	0.658	0.005	0.000
N		1,083,319			1,083,319	

Note: Fixed effects estimates (Est), standard errors (SE) and p-values based on multilevel regression models with M1URB including level of urbanization, age groups, and sex as covariate and M2URB additionally including 19 (Macro) PopGroups. Covariates with Est=0.000 are reference categories.

Table D-6: Fixed effects results from models M1URB and M2URB with indicator "Days of sickness absence"

	**Days of sickness absence**					
	**M1URB**			**M2URB**		
**Variable**	Est	SE	p-value	Est	SE	p-value
Intercept	3.584	0.021	0.000	3.451	0.024	0.000
Urban region	0.000	.	.	0.000	.	.
Semi-urban region	-0.067	0.027	0.015	-0.067	0.026	0.012
Rural region	0.011	0.027	0.686	0.022	0.027	0.413
Female	0.000	.	.	0.000	.	.
Male	0.093	0.012	0.000	0.075	0.012	0.000
Age 18-39	-0.237	0.013	0.000	-0.233	0.013	0.000
Age 40-59	0.000	.	.	0.000	.	.
Age 60-69	0.286	0.015	0.000	0.286	0.015	0.000
Age 70-79	.	.	.	.	.	.
Age >=80	.	.	.	.	.	.
P07060BB	.	.	.	0.000	.	.
P05106BB	.	.	.	0.045	0.027	0.089
P06023BB	.	.	.	0.572	0.020	0.000
P06028BB	.	.	.	0.059	0.108	0.588
P06034BB	.	.	.	0.142	0.047	0.003
P06045BB	.	.	.	0.146	0.058	0.011
P06063BB	.	.	.	0.196	0.056	0.000
P06067BB	.	.	.	-0.025	0.042	0.556
P06087BB	.	.	.	-0.077	0.043	0.071
P07010BB	.	.	.	0.049	0.055	0.376
P07025BZ	.	.	.	0.059	0.046	0.204
P07042BB	.	.	.	0.112	0.044	0.011
P07050BB	.	.	.	0.273	0.038	0.000
P07052ZB	.	.	.	0.020	0.035	0.571
P03	.	.	.	0.365	0.052	0.000
P04	.	.	.	0.145	0.067	0.030
P05	.	.	.	0.090	0.023	0.000
P06	.	.	.	0.046	0.019	0.015
P07	.	.	.	0.046	0.021	0.028
N		66,259			66,259	

Note: Fixed effects estimates (Est), standard errors (SE) and p-values based on multilevel regression models with M1URB including level of urbanization, age groups, and sex as covariate and M2URB additionally including 19 (Macro) PopGroups. Covariates with Est=0.000 are reference categories.

Table D-7: Fixed effects results from models M1URB and M2URB with indicator "Any mental health specialist contact"

	Any mental health specialist contact					
	**M1URB**			**M2URB**		
**Variable**	Est	SE	p-value	Est	SE	p-value
Intercept	-0.920	0.051	0.000	-1.211	0.053	0.000
Urban region	0.000	.	.	0.000	.	.
Semi-urban region	-0.139	0.066	0.035	-0.139	0.067	0.039
Rural region	-0.176	0.066	0.008	-0.169	0.067	0.012
Female	0.000	.	.	0.000	.	.
Male	-0.213	0.006	0.000	-0.228	0.006	0.000
Age 18-39	0.292	0.007	0.000	0.278	0.007	0.000
Age 40-59	0.000	.	.	0.000	.	.
Age 60-69	-0.466	0.006	0.000	-0.443	0.007	0.000
Age 70-79	-1.236	0.009	0.000	-1.192	0.009	0.000
Age >=80	-1.705	0.010	0.000	-1.672	0.011	0.000
P07060BB	.	.	.	0.000	.	.
P05106BB	.	.	.	0.233	0.016	0.000
P06023BB	.	.	.	1.241	0.011	0.000
P06028BB	.	.	.	0.321	0.030	0.000
P06034BB	.	.	.	0.391	0.018	0.000
P06045BB	.	.	.	-0.180	0.025	0.000
P06063BB	.	.	.	-0.086	0.025	0.001
P06067BB	.	.	.	0.923	0.021	0.000
P06087BB	.	.	.	-0.011	0.024	0.639
P07010BB	.	.	.	-0.062	0.025	0.014
P07025BZ	.	.	.	-0.041	0.021	0.055
P07042BB	.	.	.	0.107	0.022	0.000
P07050BB	.	.	.	0.486	0.022	0.000
P07052ZB	.	.	.	0.535	0.017	0.000
P03	.	.	.	0.090	0.016	0.000
P04	.	.	.	0.102	0.022	0.000
P05	.	.	.	0.229	0.010	0.000
P06	.	.	.	0.278	0.010	0.000
P07	.	.	.	0.057	0.010	0.000
N		1,083,319			1,083,319	

Note: Fixed effects estimates (Est), standard errors (SE) and p-values based on multilevel regression models with M1URB including level of urbanization, age groups, and sex as covariate and M2URB additionally including 19 (Macro) PopGroups. Covariates with Est=0.000 are reference categories.

Table D-8: Fixed effects results from models M1URB and M2URB with indicator “Outpatient psychotherapy use”

	Outpatient psychotherapy use					
	**M1URB**			**M2URB**		
**Variable**	Est	SE	p-value	Est	SE	p-value
Intercept	-1.248	0.029	0.000	-1.484	0.028	0.000
Urban region	0.000	.	.	0.000	.	.
Semi-urban region	-0.089	0.041	0.031	0.027	0.034	0.438
Rural region	-0.086	0.041	0.034	0.025	0.035	0.466
Female	0.000	.	.	0.000	.	.
Male	-0.177	0.006	0.000	-0.175	0.006	0.000
Age 18-39	0.076	0.008	0.000	0.078	0.008	0.000
Age 40-59	0.000	.	.	0.000	.	.
Age 60-69	-0.241	0.007	0.000	-0.243	0.007	0.000
Age 70-79	-0.287	0.008	0.000	-0.289	0.008	0.000
Age >=80	-0.496	0.008	0.000	-0.503	0.008	0.000
P07060BB	.	.	.	0.000	.	.
P05106BB	.	.	.	0.272	0.016	0.000
P06023BB	.	.	.	0.332	0.012	0.000
P06028BB	.	.	.	0.140	0.028	0.000
P06034BB	.	.	.	0.293	0.018	0.000
P06045BB	.	.	.	0.052	0.021	0.014
P06063BB	.	.	.	0.108	0.022	0.000
P06067BB	.	.	.	0.139	0.025	0.000
P06087BB	.	.	.	0.128	0.025	0.000
P07010BB	.	.	.	0.128	0.023	0.000
P07025BZ	.	.	.	0.088	0.020	0.000
P07042BB	.	.	.	0.110	0.022	0.000
P07050BB	.	.	.	0.068	0.025	0.007
P07052ZB	.	.	.	0.113	0.019	0.000
P03	.	.	.	0.095	0.015	0.000
P04	.	.	.	0.318	0.019	0.000
P05	.	.	.	0.149	0.011	0.000
P06	.	.	.	0.161	0.010	0.000
P07	.	.	.	0.100	0.011	0.000
N		1,083,319			1,083,319	

Note: Fixed effects estimates (Est), standard errors (SE) and p-values based on multilevel regression models with M1URB including level of urbanization, age groups, and sex as covariate and M2URB additionally including 19 (Macro) PopGroups. Covariates with Est=0.000 are reference categories.
