# Supplementary material for: Regional variation in healthcare utilization among patients with depression in Germany: a multilevel analysis with PopGrouper-based multimorbidity adjustment
Source: Res Health Serv Reg. 2026 Jun 9;5:8. doi: 10.1007/s43999-026-00092-6 (PMC13250020; doi:10.1007/s43999-026-00092-6)
Supplement: Supplementary file 5 — Supplementary Material 5 [file 43999_2026_92_MOESM5_ESM.pdf]

Supplement E: Effect ratios based on multilevel regression results presenting association between (Macro)PopGroups and depression-related healthcare utilization

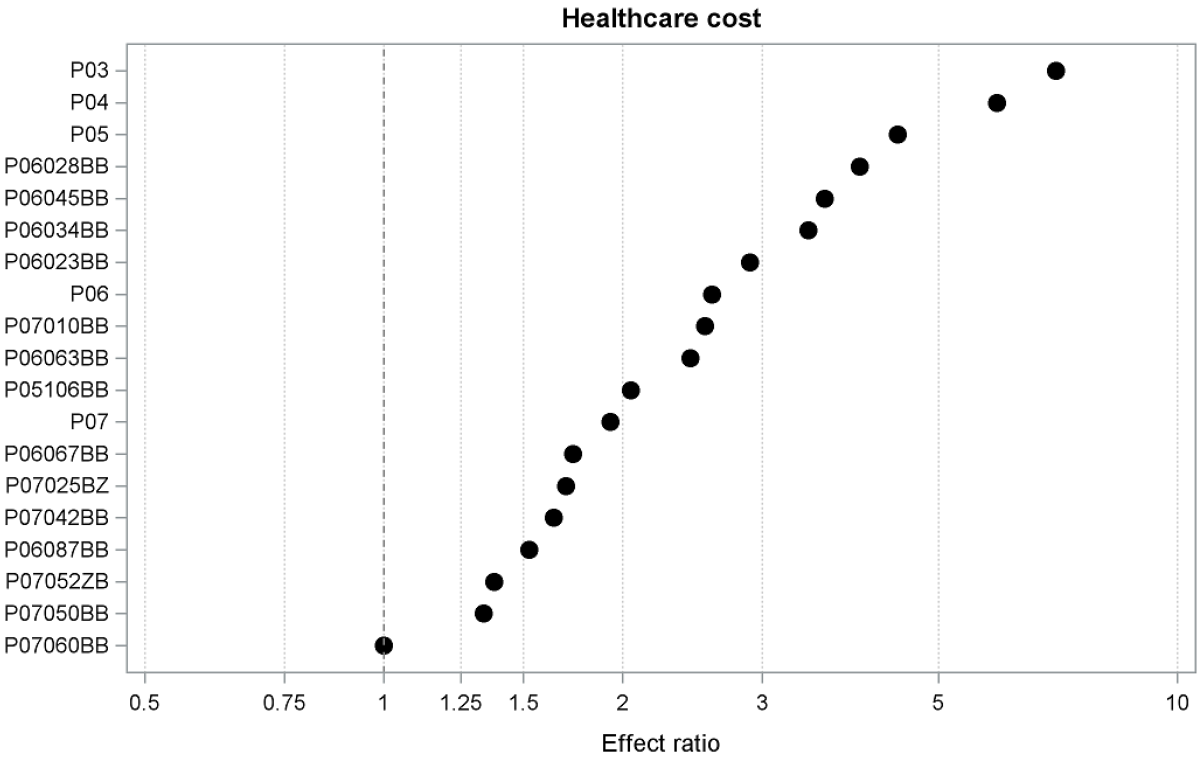

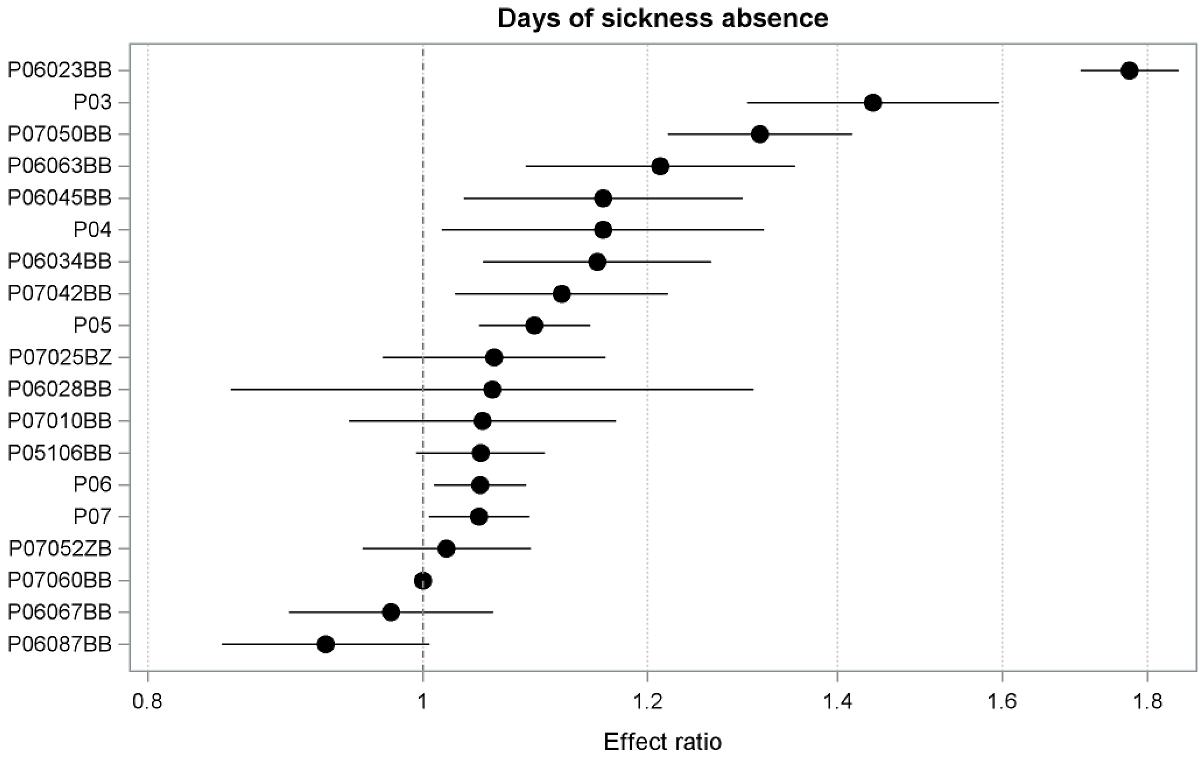

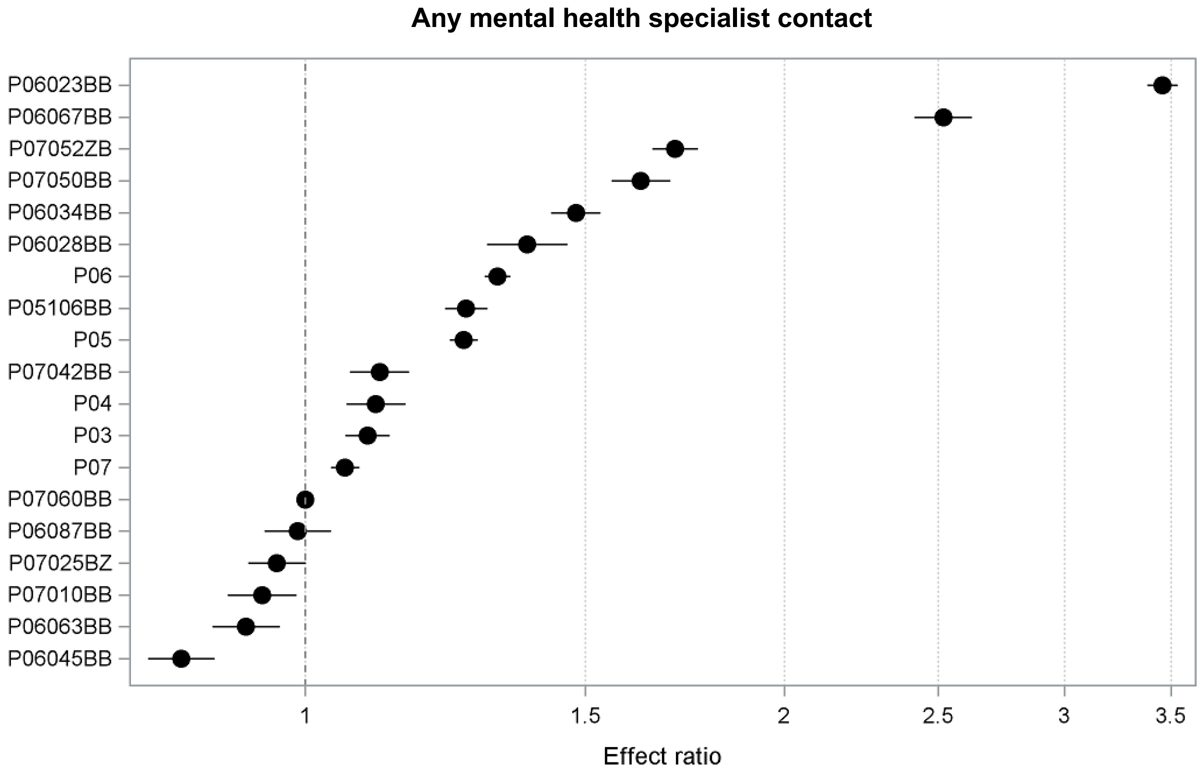

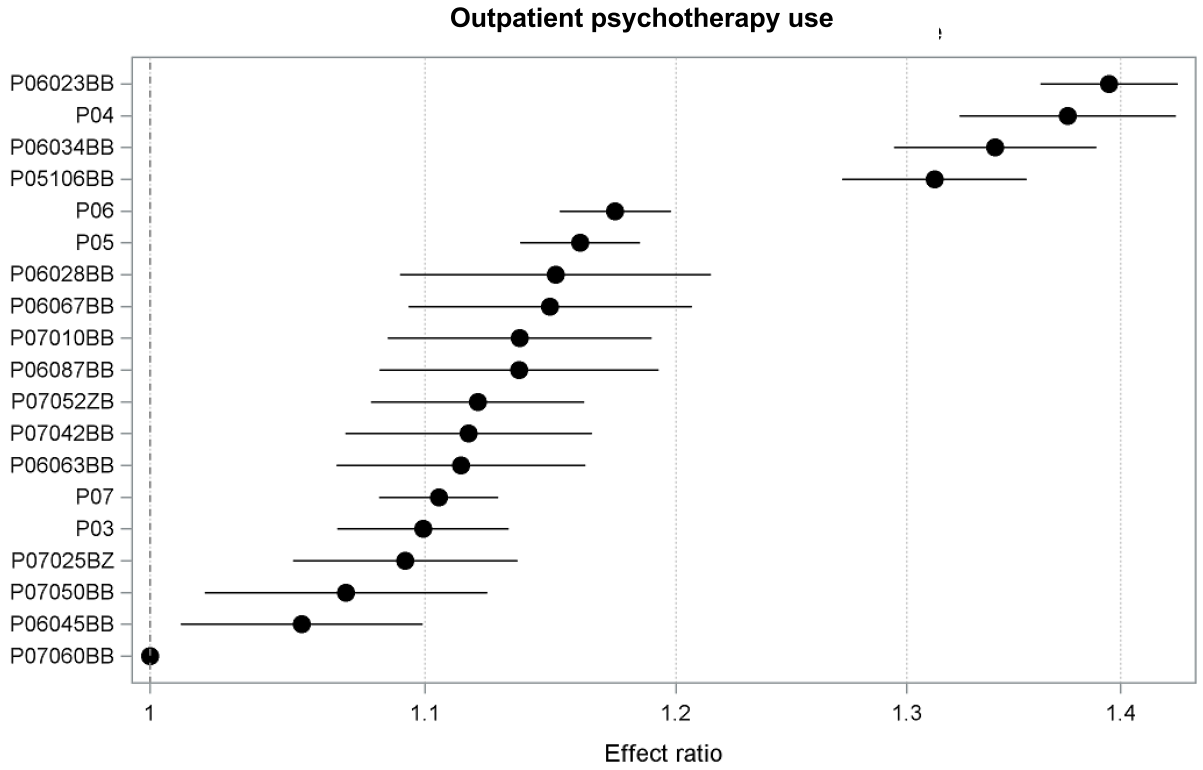

Note: Points represent effect ratios (exp(β)) from multilevel regression model M2DEP adjusted for deprivation, age, sex and (Macro)PopGroup. Error bars indicate 95% confidence intervals. PopGroup P07060BB is the reference category. (Macro)PopGroup names are referenced in Table 2 of the main manuscript. Values above 1 indicate higher levels of the outcome compared to the reference, while values below 1 indicate lower levels. The vertical dashed line indicates no difference (effect ratio = 1).
