# Supplementary material for: Regional variation in healthcare utilization among patients with depression in Germany: a multilevel analysis with PopGrouper-based multimorbidity adjustment
Source: Res Health Serv Reg. 2026 Jun 9;5:8. doi: 10.1007/s43999-026-00092-6 (PMC13250020; doi:10.1007/s43999-026-00092-6)
Supplement: Supplementary file 6 — Supplementary Material 6 [file 43999_2026_92_MOESM6_ESM.pdf]

Supplement F: Region-level random effects based on multilevel regression results

Displayed are region-specific random intercepts from fully adjusted multilevel models (M2DEP). Random effects are shown on the exponentiated scale and can be interpreted as region-specific relative deviations from the overall mean. Values above 1 indicate higher levels of the outcome compared to the national average, while values below 1 indicate lower levels. The vertical dashed line represents the overall mean (effect ratio = 1).

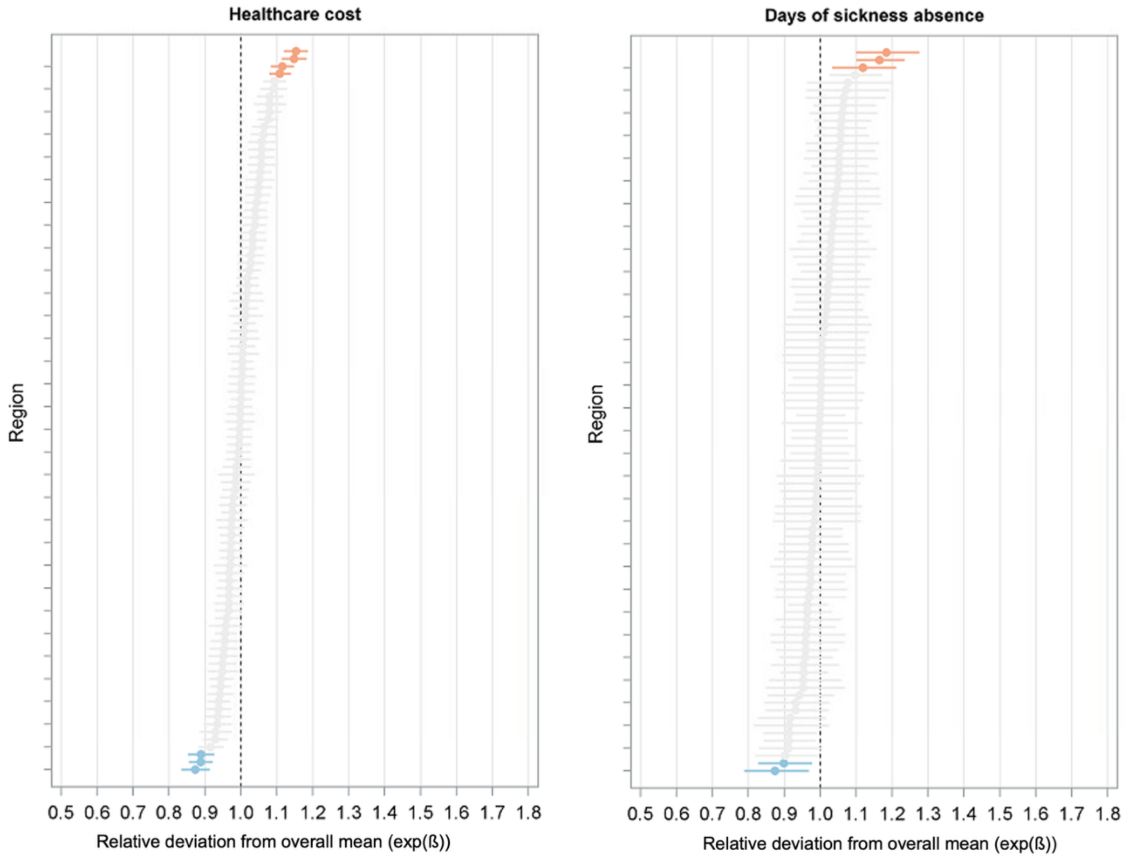

Figure F-1: Region-level random effects for healthcare cost and sickness absence after adjustment for socioeconomic deprivation and patient-level characteristics

Note: Points represent estimated random intercepts from model M2DEP, with horizontal lines indicating 95% confidence intervals. Random effects are shown on the exponentiated scale and can be interpreted as region-specific relative deviations from the overall mean. Regions are ordered by the magnitude of the random effects.

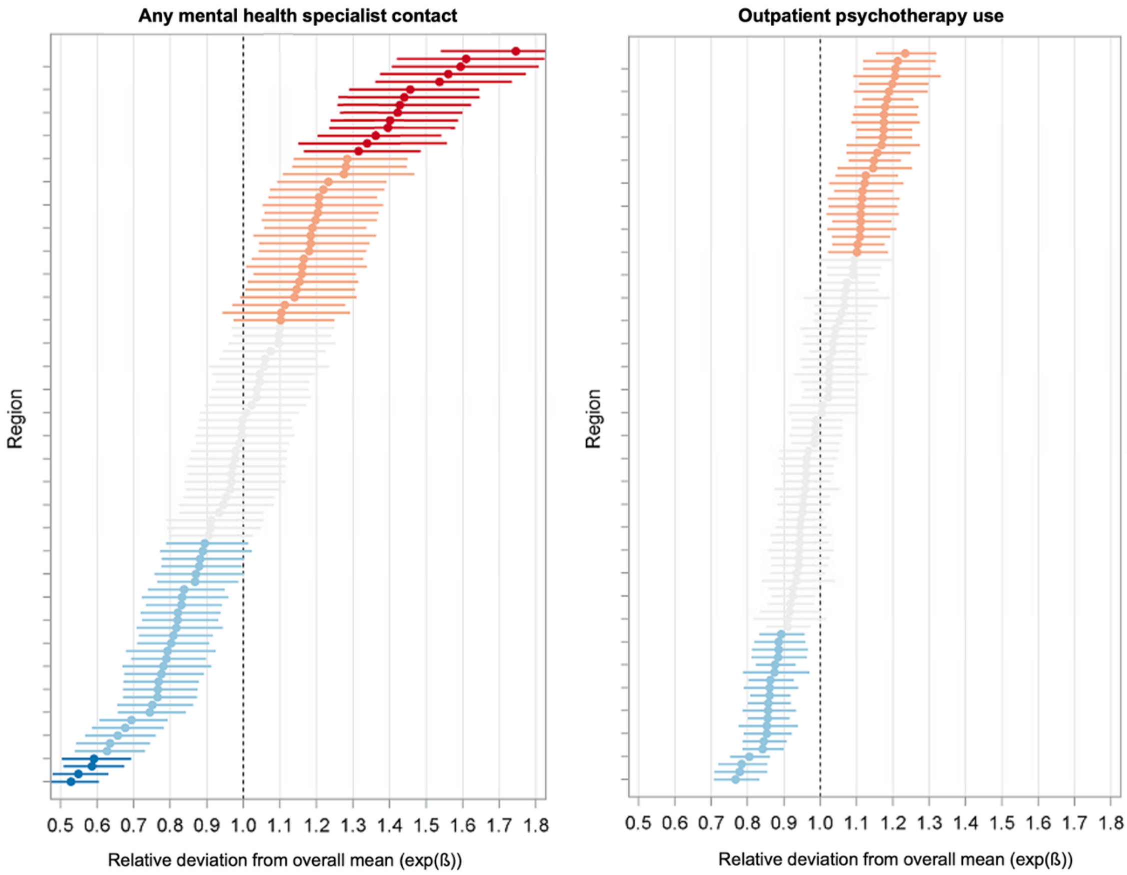

Figure F-2: Region-level random effects for psychotherapy service utilization after adjustment for socioeconomic deprivation and patient-level characteristics

Note: Points represent estimated random intercepts from model M2DEP, with horizontal lines indicating 95% confidence intervals. Random effects are shown on the exponentiated scale and can be interpreted as region-specific relative deviations from the overall mean. Regions are ordered by the magnitude of the random effects.

Residual regional variation was small for healthcare costs and modest for days of sickness absence, with most regions close to the overall mean. In contrast, substantial unexplained heterogeneity persisted for any outpatient mental health specialist contact, while outpatient psychotherapy use showed moderate variation.
